# Supramolecular Chiral Assemblies from Benzoselenadiazole‐Alanine‐Acylhydrazone Conjugates Enable Cardioprotection

**DOI:** 10.1002/advs.77953

**Published:** 2026-09-27

**Authors:** Jinkui Teng, Renjing Yang, Yu Du, Jingzhong Duan, Yiming Xu, Zhihua He, Guanghui Wang, Wenjing Tian, Aijie Liu, Haifeng Chen, Yun‐Bao Jiang, Xiaosheng Yan

**Affiliations:** ^1^ State Key Laboratory of Vaccines for Infectious Diseases Xiang an Biomedicine Laboratory Fujian Provincial Key Laboratory of Innovative Drug Target Research School of Pharmaceutical Sciences Xiamen University Xiamen Fujian China; ^2^ Zhongshan Hospital Xiamen University Xiamen Fujian China; ^3^ Jiujiang Research Institute of Xiamen University Jiujiang Jiangxi China; ^4^ Department of Chemistry College of Chemistry and Chemical Engineering The MOE Key Laboratory of Spectrochemical Analysis and Instrumentation Xiamen University Xiamen China

**Keywords:** benzoselenadiazole, cardioprotection, chalcogen bonding, doxorubicin, supramolecular chiral assembly

## Abstract

Integrating structural order, permanent porosity, aqueous stability, and biological activity within a single supramolecular platform remains a central challenge in biomaterials science. Herein, we report a C_3_‐symmetric conjugate (**TASe**) that unites benzoselenadiazole, alanine, and acylhydrazone motifs, each serving dual roles as both supramolecular interaction sites and bioactive pharmacophores. This multifunctional design enables synergistic noncovalent assembly through cooperative chalcogen and hydrogen bonds, which direct the formation of a porous crystalline framework. In aqueous media, the same molecular modules drive self‐assembly into uniform chiral nanospheres. The resulting assemblies efficiently protect cardiomyocytes from doxorubicin‐induced injury by scavenging reactive oxygen species, reducing oxidative DNA damage, and attenuating ERK activation, without compromising anticancer efficacy. By embedding both assembly‐directing and therapeutic functions within a single molecular scaffold, this work provides a generalizable strategy for constructing biologically active supramolecular materials with coordinated structural order and function.

## Graphical abstract

The C_3_‐symmetric conjugate **TASe**, which integrates benzoselenadiazole, alanine, and acylhydrazone modules, self‐assembles into a chalcogen‐/hydrogen‐bonded porous framework in the solid state and uniform chiral nanospheres in aqueous media. These assemblies protect cardiomyocytes from doxorubicin‐induced oxidative injury by scavenging intracellular reactive oxygen species, while preserving the antitumor efficacy of the drug.

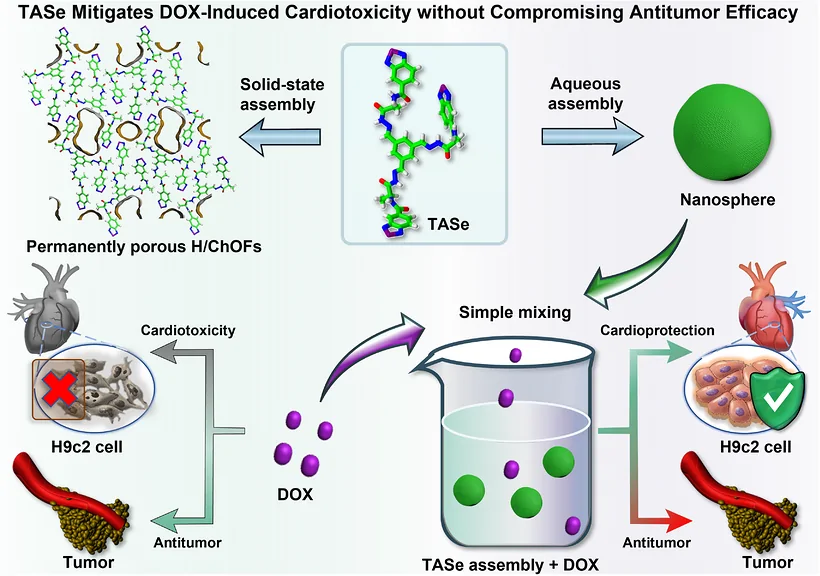

## 1 Introduction

Supramolecular materials constructed from chemically defined organic molecules have attracted considerable attention because they enable the translation of molecular‐level structural information into ordered architectures and emergent functions through noncovalent interactions [1]. Among the diverse interaction motifs available for supramolecular design, hydrogen bonding remains one of the most powerful and widely used tools owing to its directionality, reversibility, and synthetic accessibility [2]. In parallel, chalcogen bonding has recently emerged as an important σ‐hole interaction for molecular recognition [3], crystal engineering, and functional materials construction [4, 5, 6, 7]. Selenium‐ and tellurium‐containing heteroarenes, for instance, can engage electron‐rich oxygen, nitrogen, or halide acceptors through directional chalcogen bonds, thereby reinforcing molecular packing or modulating host‐guest binding [8, 9]. The cooperative use of hydrogen bonding and chalcogen bonding therefore provides an attractive strategy for developing supramolecular systems with enhanced structural precision, hierarchical organization, and environmental robustness. However, realizing such synergy within a single molecular platform that also retains intrinsic bioactivity requires careful selection of complementary functional modules.

Despite these advances, integrating multiple noncovalent interactions into a single molecular platform that simultaneously exhibits robust self‐assembly and useful biological activity remains challenging. Strong assembly motifs, such as highly π‐conjugated aromatic frameworks and strongly hydrogen‐bonded crystalline domains, can enhance intermolecular interactions, molecular ordering, and structural stability, but often at the expense of aqueous dispersibility, dynamic adaptability, and biocompatibility [10, 11, 12, 13]. Conversely, many bioactive small molecules, including phenolic antioxidants, redox‐active heterocycles, and clinically used cytoprotective agents, exhibit clear pharmacological activity as discrete molecular species but generally lack the persistent supramolecular cohesion needed to form stable nanostructures in aqueous media [14, 15, 16]. This structure‐function mismatch is particularly evident in combination therapy, where one component must reduce the dose‐limiting toxicity of a partner drug while preserving, or even enhancing, its therapeutic efficacy [17, 18, 19, 20]. Therefore, there is an urgent need to develop function‐integrated molecular platforms in which the same structural motifs can direct ordered supramolecular assembly through cooperative noncovalent interactions, maintain stable molecular association with therapeutic agents, and simultaneously endow the system with intrinsic protective or synergistic bioactivity.

**SCHEME 1:**
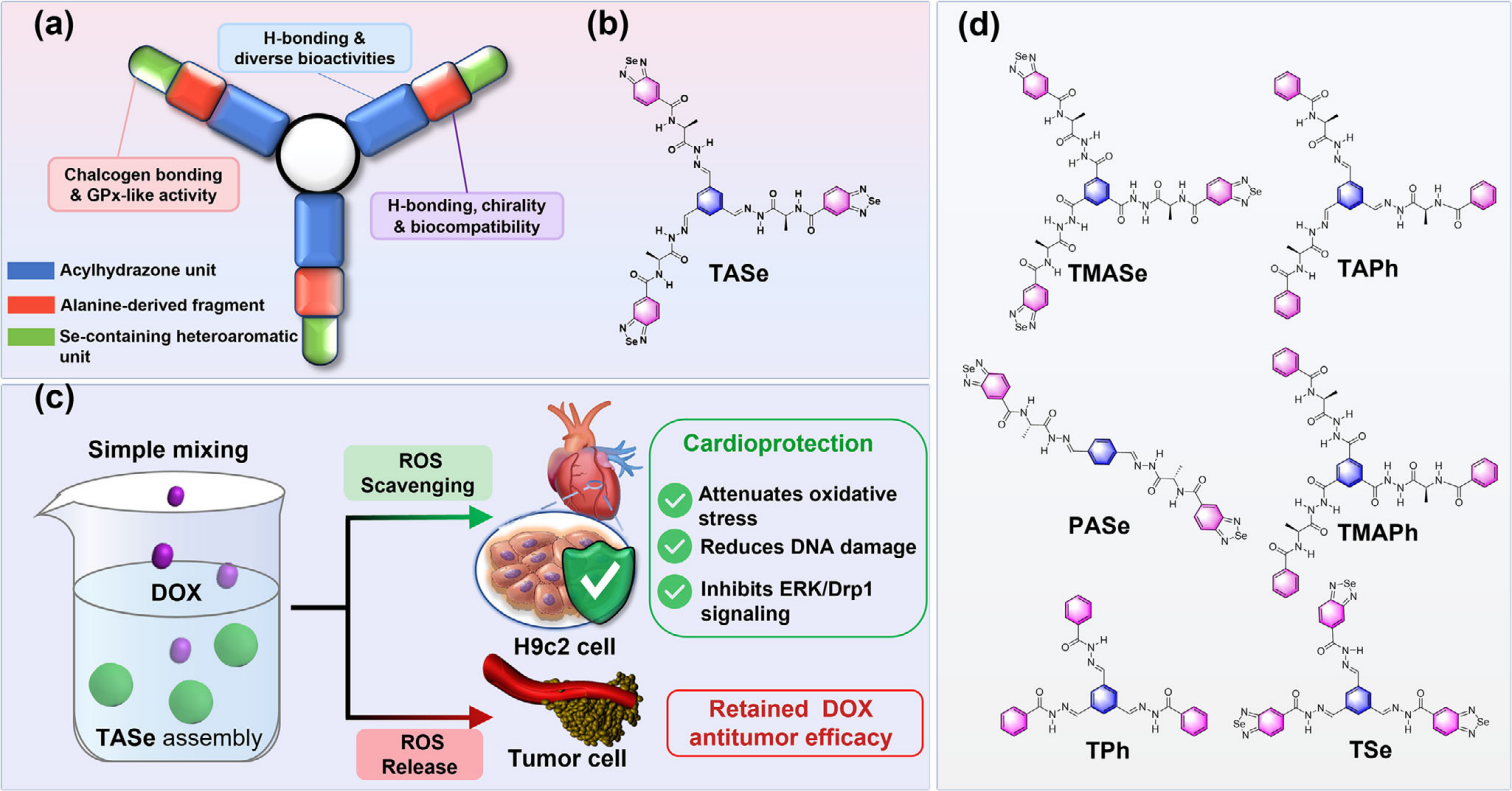
Molecular design and cardioprotective bioactivity of **TASe**. (a) Schematic illustration of the **TASe** building block, which comprises a tripodal phenyl core, acylhydrazone linkers, alanine‐derived segments, and terminal benzoselenadiazole units. (b) Chemical structure of **TASe**. (c) Schematic representation of the biological functions of **TASe** assemblies: the assemblies attenuate DOX‐induced oxidative stress, suppress DNA damage, and inhibit ERK/Drp1‐mediated cardiotoxic signaling in cardiomyocytes, while preserving the antitumor efficacy of DOX against cancer cells. (d) Chemical structures of control molecules designed to dissect the functional contributions of key structural motifs in **TASe**, including the hydrazone linkage, benzoselenadiazole unit, alanine segment, and tripodal architecture.

A rational approach to addressing this challenge is to incorporate several interaction‐capable and bioactive motifs into one chemically defined scaffold (Scheme 1a). Acylhydrazone units integrate multiple hydrogen‐bonding sites and dynamic covalent character [21], alongside diverse bioactivities including antioxidant and anti‐inflammatory effects [22, 23, 24, 25, 26, 27]. Alanine‐derived fragments introduce additional hydrogen‐bond donors/acceptors, stereochemical information, and biocompatibility [28, 29, 30]. Selenium‐containing heteroaromatic units, beyond their ability to participate in directional chalcogen bonding [31], have been widely studied for selenium‐based reactive oxygen species (ROS)‐regulating behavior [32]. Thus, these three modules are uniquely positioned to serve dual roles as both supramolecular recognition elements and bioactive pharmacophores [33, 34, 35].

Inspired by the antioxidant function of selenium‐containing glutathione peroxidase (GPx) [36, 37, 38], we reasoned that incorporating selenadiazole units into a supramolecular system could provide selenium‐centered antioxidant motifs for protecting cardiomyocytes against oxidative injury (Scheme 1a). Doxorubicin (DOX) is a first‐line anthracycline chemotherapeutic agent widely used to treat breast cancer. However, its clinical utility is severely restricted by cumulative and dose‐dependent cardiac injury^[^ ^39^ ^]^. Mechanistically, this cardiotoxicity is closely associated with excessive ROS generation and downstream oxidative damage to DNA, mitochondria, and cellular signaling pathways [40, 41, 42]. Although antioxidant interventions have been explored to alleviate these adverse effects, conventional antioxidants often suffer from rapid clearance, limited intracellular retention, and poorly defined structure‐activity relationships, and in some cases may even compromise the anticancer efficacy of DOX [43, 44, 45]. In this context, a supramolecular system that integrates redox‐active selenium motifs with stable aqueous assembly could offer distinct advantages.

Herein, we report **TASe**, a C_3_‐symmetric conjugate of benzoselenadiazole, alanine, and acylhydrazone (Scheme 1b), which exemplifies the aggregatism paradigm, where emergent functions arise from aggregate systems beyond the capabilities of isolated molecules [46]. Single‐crystal x‐ray diffraction reveals that **TASe** forms an extended chalcogen‐/hydrogen‐bonded organic framework, in which intermolecular N─H···O hydrogen bonds cooperate with directional Se···N chalcogen bonds to generate a highly crystalline and robust supramolecular structure with permanent porosity. In aqueous media, **TASe** self‐assembles into uniform chiral nanospheres with long‐term colloidal stability. Importantly, **TASe** protects H9c2 cardiomyocytes from DOX‐induced injury by suppressing ROS accumulation, reducing oxidative DNA damage, and attenuating aberrant ERK activation, while preserving the anticancer activity of DOX (Scheme 1c). This work demonstrates that embedding both assembly‐directing and bioactive functions within a single molecular platform provides a viable route to supramolecular materials with integrated structural order and therapeutic function.

## 2 Results and Discussion

### 2.1 Molecular Synthesis and Characterization

***L*‐TASe** and ***D*‐TASe** were synthesized as a C_3_‐symmetric conjugate of benzoselenadiazole, alanine, and acylhydrazone (Scheme 1). The synthesis was achieved efficiently by condensation of our previously developed benzoselenadiazole‐alanine hydrazide [47] with 1,3,5‐benzenetricarboxaldehyde in an ethanol/water (v/v = 1:2) mixed solvent. The target product was obtained in 90% yield after simple washing with ethanol.

Interestingly, the room‐temperature ^1^H NMR spectrum of ***L*‐TASe** in DMSO‐*d*_6_ displayed two sets of proton signals with an integral ratio close to 1:1 (Figure S1a). The downfield signals at 11.6–11.7 ppm are assigned to the N–H protons of the hydrazone moieties, while those at 8.9–9.0 ppm correspond to the amide N–H protons. Meanwhile, the upfield signals in the range of 4.5–5.4 ppm are attributed to the protons on the chiral carbon of alanine moieties. The observed signal splitting is attributed to the coexistence of *cis* and *trans* conformations about the amide bonds within the acylhydrazone motifs [48].

### 2.2 Single‐Crystal Structure

**FIGURE 1:**
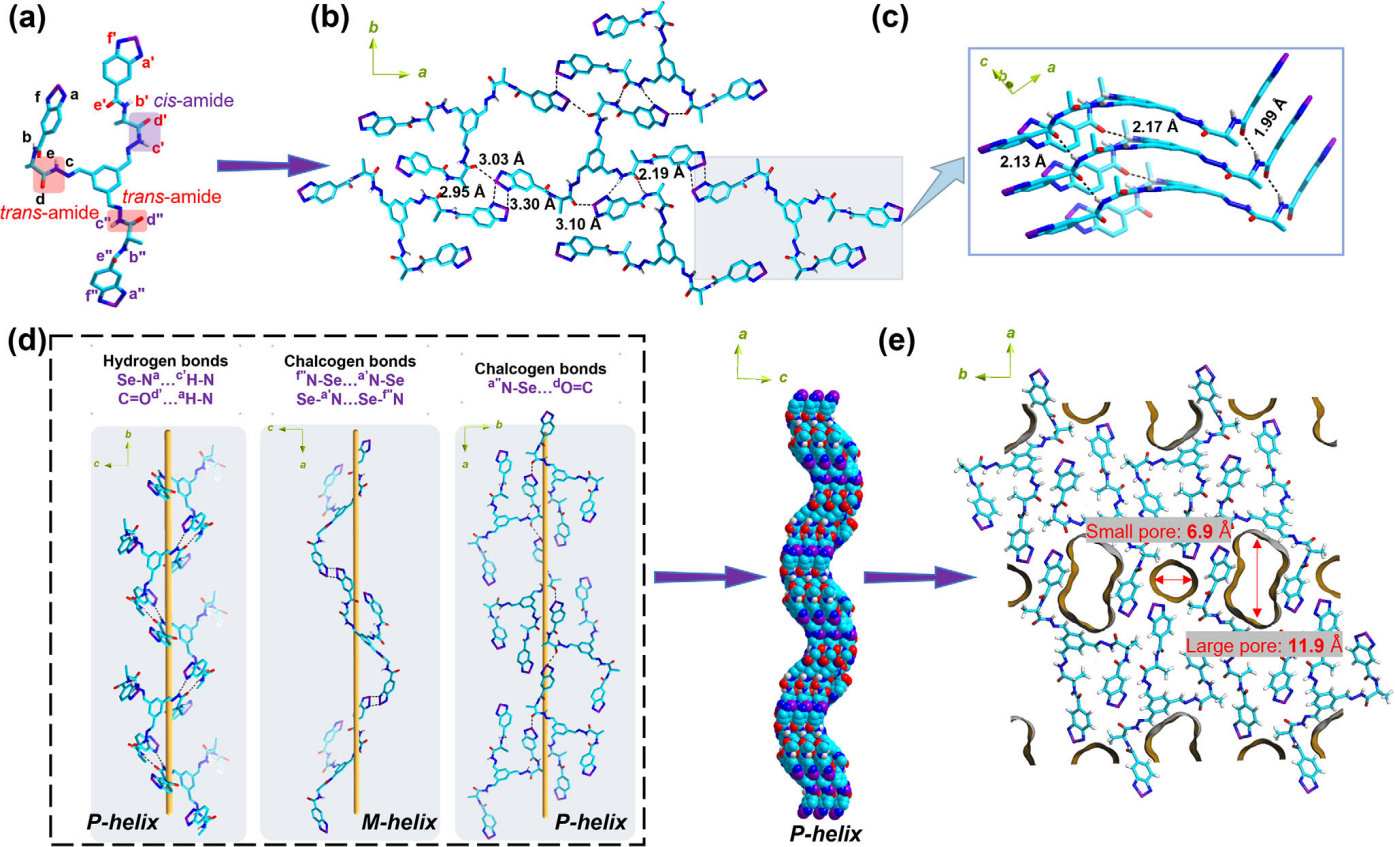
Supramolecular interactions, crystal packing, and hierarchical helical porous assembly of ***L*‐TASe**. (a) Single‐molecule crystal structure of ***L*‐TASe**, showing three conformationally distinct arms with one *cis* and two *trans* amide conformations. (b) Crystal‐packing view within the *ab* plane, showing cooperative intermolecular chalcogen bonds (Se···N, Se···O) and hydrogen bonds (N─H···O, N─H···N). (c) Layered packing arrangement viewed along the *b*‐axis, illustrating the undulated supramolecular architecture stabilized by a triple N–H···O hydrogen‐bonding network. (d) Schematic representation of three distinct helical motifs (*P*‐, *M*‐, and *P*‐type) generated along different crystallographic axes through cooperative chalcogen and hydrogen bonds. The space‐filling model on the right shows the dominant *P*‐type supramolecular helix. (e) Hierarchical porous architecture viewed along the *c*‐axis, showing one‐dimensional channels with pore apertures of approximately 6.9 and 11.9 Å.

Single crystals of ***L/D*‐TASe** were cultivated by slow diffusion of water vapor into a DMSO solution of ***L/D*‐TASe** at 75 °C (Figure S2). Single‐crystal x‐ray diffraction (SCXRD) analysis revealed that ***L*‐TASe** crystallizes in the *P*2_1_2_1_2 space group (Table S1), in which the intrinsic C_3_‐symmetry of the molecule is not retained. One ***L*‐TASe** molecule contains three conformationally distinct arms: the amide bond of one acylhydrazone arm adopts a *cis*‐conformation, while the other two arms adopt *trans*‐conformations (Figure 1a). This solid‐state conformation is fully consistent with the *cis*/*trans* signal splitting observed in the solution‐phase ^1^H NMR spectrum (Figure S1), confirming that the conformational heterogeneity persists from solution to the crystalline state.

Within the *ab* plane, cooperative intermolecular N─H···O, N─H···N hydrogen bonds and Se···N, Se···O chalcogen bonds link one ***L*‐TASe** molecule to five neighboring molecules, generating an undulating layered architecture (Figure 1b). Along the *c*‐axis, these layers are further stabilized by a triple N─H···O hydrogen‐bonding network, which reinforces the long‐range structural integrity of the framework (Figure 1c). Notably, all three designed structural modules participate in these intermolecular interactions. The acylhydrazone and alanine units provide N–H and C = O groups for hydrogen bonding, while the benzoselenadiazole termini engage in Se‐centered chalcogen bonding (Table S2).

Beyond the layered organization, the interplay of hydrogen and chalcogen bonds generates three distinct helical motifs along different crystallographic axes, assigned as *P*‐, *M*‐, and *P*‐helices. These motifs collectively assemble into a long‐range supramolecular helical framework dominated by *P*‐type helicity (Figure 1d). The resulting helical framework further packs into a three‐dimensional hierarchical porous architecture featuring two distinct pore sizes of approximately 6.9 and 11.9 Å in diameter (Figure 1e). ***D*‐TASe** displays mirror‐related helical assembly, forming *M*‐, *P*‐, and *M*‐type helices and an overall *M*‐type supramolecular helical framework (Figures S3 and S4).

### 2.3 Solid‐State Stability, Chirality, and Porosity

**FIGURE 2:**
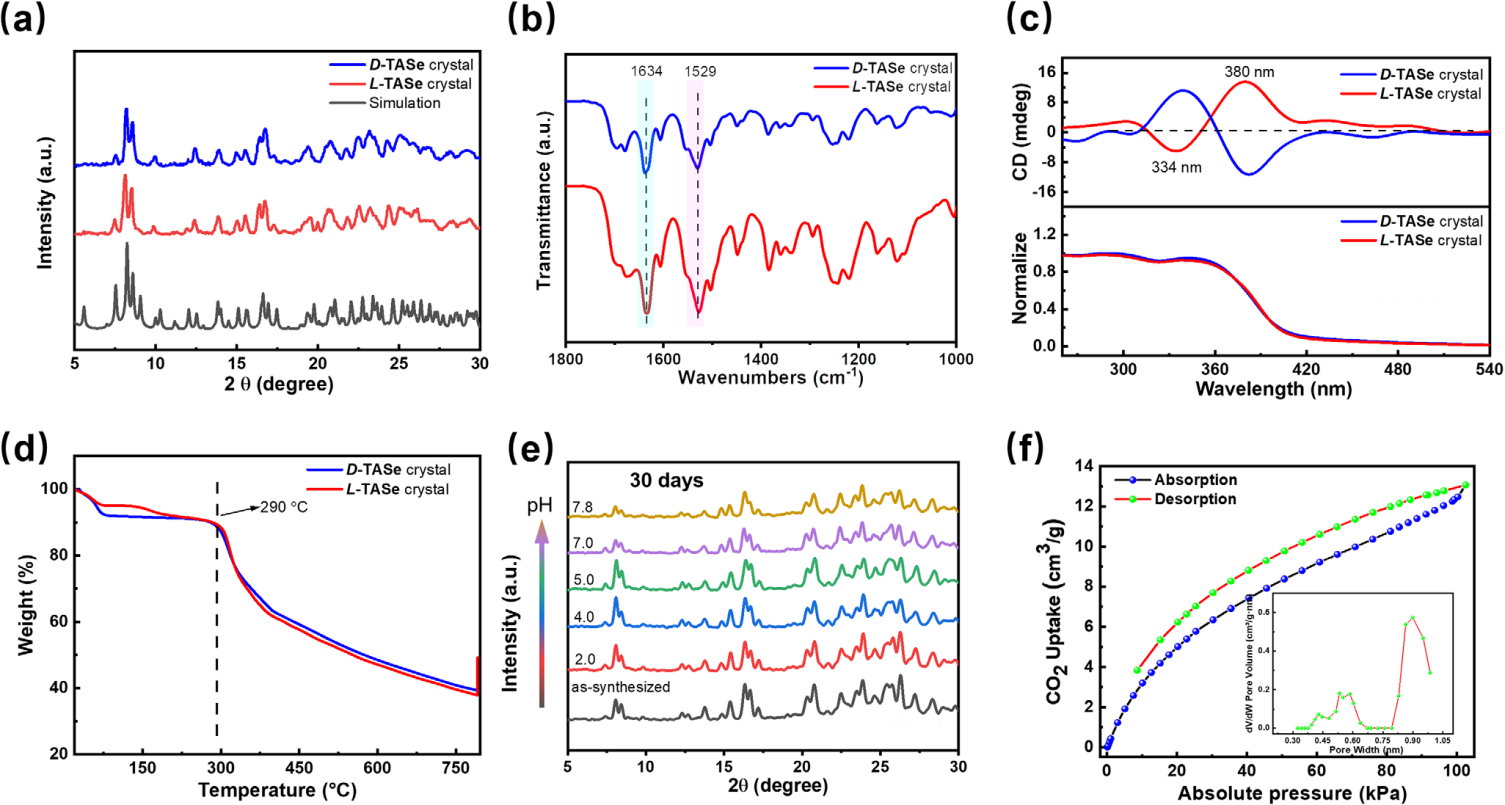
Solid‐state characterization of ***L*‐TASe** and ***D*‐TASe** crystals. (a) Experimental PXRD patterns of ***L*‐TASe** and ***D*‐TASe** and simulated pattern from SCXRD data, confirming phase purity and crystallinity. (b) FTIR spectra of ***L*‐TASe** and ***D*‐TASe**, showing amide I and amide II vibrations. (c) Solid‐state CD and absorption spectra of ***L*‐TASe** and ***D*‐TASe**. Mirror‐image Cotton effects confirm their enantiomeric relationship. (d) TGA curves of ***L*‐TASe** and ***D*‐TASe**, showing thermal stability up to 290°C. (e) PXRD patterns of ***L*‐TASe** after 30‐day immersion in aqueous media at pH 2.0 – 7.8, demonstrating chemical robustness. (f) CO_2_ adsorption‐desorption isotherms of ***L*‐TASe** at 273 K. Inset: pore size distribution, revealing microporous features.

The phase purity and structural integrity of the bulk materials were confirmed by powder x‐ray diffraction (PXRD). The experimental patterns of as‐synthesized ***L*‐TASe** and ***D*‐TASe** are nearly identical and match well with the pattern simulated from SCXRD data, confirming that the bulk samples retain the crystallographic structure (Figure 2a). The well‐resolved low‐angle diffraction peaks further indicate the preservation of the long‐range ordered porous framework (Figure S5). The PXRD pattern remained unchanged after thermal vacuum treatment (Figure S6), demonstrating excellent structural stability of the framework.

Fourier‐transform infrared spectroscopy (FTIR) spectra of ***L*‐TASe** and ***D*‐TASe** crystals showed nearly identical absorption profiles, consistent with their enantiomeric relationship (Figure 2b). The bands at ∼1634 and ∼1529 cm^−1^ are assigned to amide I (C═O stretching) and amide II (N─H bending coupled with C─N stretching) vibrations, respectively [49, 50]. The absence of new bands or peak shifts indicates that chirality inversion does not alter the crystal packing or local bonding environment. Solid‐state circular dichroism (CD) spectroscopy further revealed mirror‐image Cotton effects for the two enantiomers, with opposite signs at 380 and 334 nm, confirming the successful transfer of chirality from the molecular building blocks to the bulk framework (Figure 2c). The absorption maximum at ∼345 nm is slightly offset from the CD‐active wavelengths, indicating exciton coupling between chromophores arranged in a chiral supramolecular architecture.

The robustness of the chalcogen‐/hydrogen‐bonded framework was further evaluated in terms of thermal and chemical stability. Thermogravimetric analysis (TGA) showed negligible weight loss up to ∼290°C for both enantiomers, followed by sharp decomposition above this temperature (Figure 2d). Given the potential acid lability of hydrazone bonds, the aqueous stability of ***L*‐TASe** crystals was examined over a pH range of 2.0–7.8 for 30 days. Both PXRD patterns and FTIR spectra remained essentially unchanged after treatment across all pH conditions (Figure 2e and Figure S7), demonstrating the remarkable chemical robustness of the crystalline framework and supporting its suitability for physiological applications.

The permanent porosity of ***L*‐TASe** crystals was assessed by CO_2_ adsorption‐desorption isotherms at 273 K (Figure 2f). The isotherm displayed a type‐I profile with a maximum uptake of ∼13 cm^3^ g^−1^ at 100 kPa, and the nearly overlapping adsorption and desorption branches indicated reversible gas adsorption without framework collapse. The pore size distribution centered at ∼0.5 and 0.9 nm is consistent with the microporous structure observed by SCXRD. Notably, chalcogen‐bonded organic frameworks (ChOFs) with permanent porosity remain exceedingly rare, and reports of such materials based on selenium‐centered chalcogen bonds are even more limited [9, 51]. To the best of our knowledge, **TASe** represents one of the few examples of a permanently porous framework assembled through cooperative Se···N chalcogen bonds, highlighting the viability of chalcogen bonding as a powerful and underutilized tool for constructing robust porous supramolecular architectures. Collectively, these results confirm the high crystallinity, excellent thermal and chemical stability, and permanent microporosity of the ***L***‐**TASe** framework.

### 2.4 Solution‐State Self‐Assembly and Colloidal Stability

**FIGURE 3:**
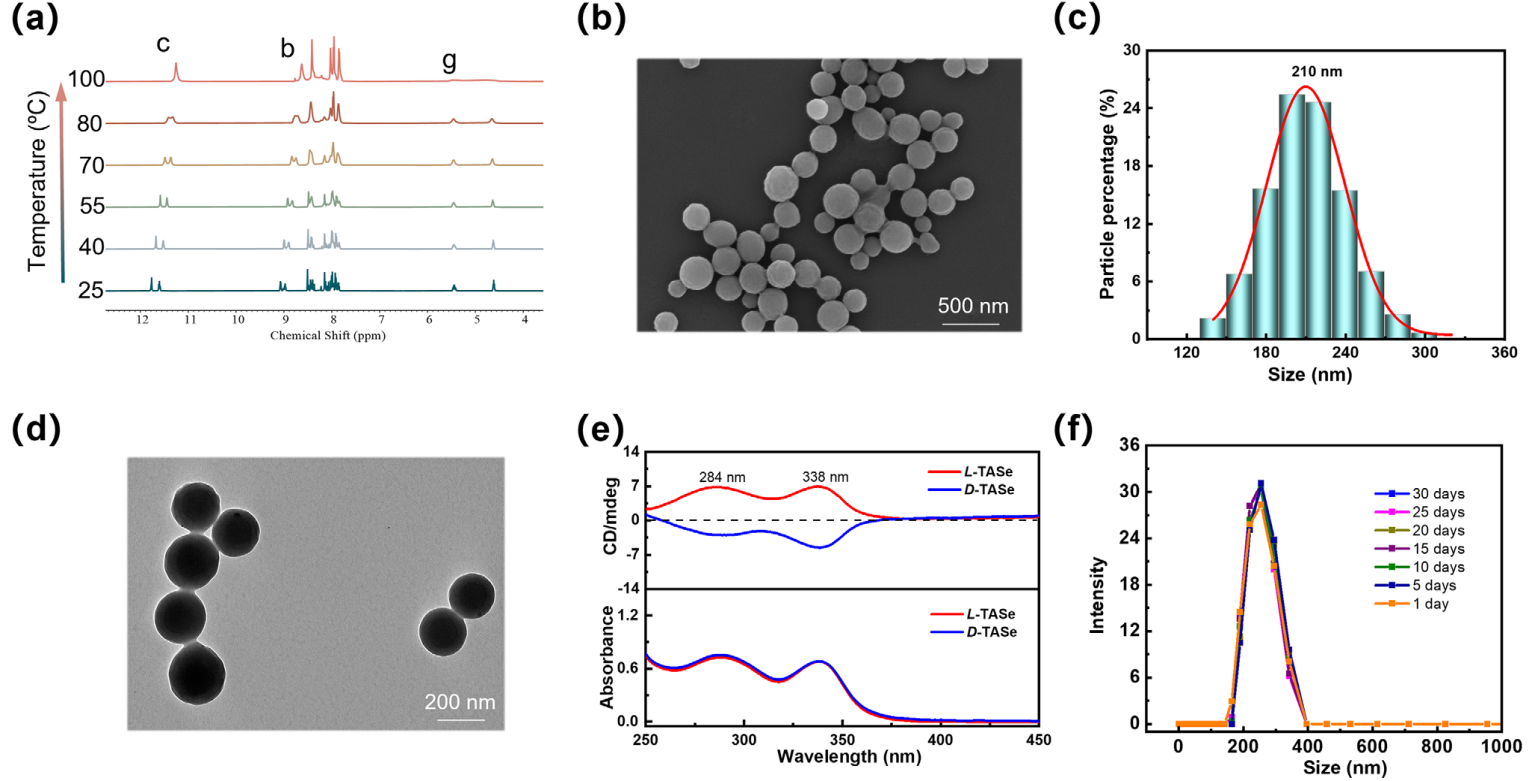
Characterization of solution self‐assembly behavior of ***L*‐TASe**. (a) Variable‐temperature ^1^H NMR spectra of ***L*‐TASe** in DMSO‐*d*_6_ over the range of 25°C–100°C. (b) SEM image of ***L*‐TASe** assemblies, showing uniform spherical morphology. (c) Size distribution of the assemblies from SEM analysis, with an average diameter of 210 nm. (d) TEM image confirming the spherical morphology of ***L*‐TASe** assemblies. (e) CD and absorption spectra of ***L*‐TASe** and ***D*‐TASe** assemblies, displaying mirror‐image Cotton effects. (f) Time‐dependent DLS profiles of ***L*‐TASe** assemblies over 30 days, demonstrating colloidal stability.

The solution‐state behavior of **TASe** was investigated following solid‐state structural characterization. Variable‐temperature ^1^H NMR spectroscopy in DMSO‐*d_6_* revealed that upon heating from 25°C to 100°C, the hydrazone and amide proton resonances progressively broadened and coalesced, whereas signals at the chiral center remained split into two sets, indicating restricted amide bond rotation rather than C = N isomerization (Figure 3a). This behavior is consistent with the *cis*/*trans* amide conformations observed in the crystal structure.

Upon dispersion in aqueous media, ***L*‐TASe** readily formed colloidal aggregates, as evidenced by a clear Tyndall effect at concentrations as low as 1–10 µM (Figure S8). The aggregated state was further supported by concentration‐dependent fluorescence spectroscopy. Excitation at ∼350 nm gave rise to a pronounced emission at 705 nm, which we attribute to scattering from the nanoassemblies rather than molecular fluorescence (Figure S9). The emission intensity increased with concentration up to 20 µM and decreased at higher concentrations, likely due to enhanced aggregation.

Dynamic light scattering (DLS) measurements showed that ***L***‐**TASe** formed well‐dispersed nanoassemblies with narrow size distributions across 5°C40 µM, with hydrodynamic diameters ranging from 240 to 280 nm (Figure S10). Scanning electron microscopy (SEM) and transmission electron microscopy (TEM) images confirmed uniform spherical morphologies with an average diameter of ∼210 nm, consistent with the DLS results (Figure 3b–d and Figures S11 and S12). The homogeneous electron contrast observed by TEM indicated compact nanospheres without hollow interiors.

Supramolecular chirality of the aqueous nanoassemblies was examined by CD spectroscopy. ***L*‐TASe** and ***D*‐TASe** displayed mirror‐image Cotton effects with nearly identical absorption spectra, confirming that the two enantiomers adopt opposite chiral packing arrangements while sharing the same electronic transitions (Figure 3e). The CD peaks at 338 and 284 nm feature the same sign and lack exciton‐coupled characteristics. These signals arise from chirality transfer from the alanine stereocenters to the supramolecular level and are encoded in the internal molecular packing rather than in macroscopic helical morphology. This assignment is further supported by linear dichroism (LD) measurements, which showed negligible LD signals for ***L*‐TASe** in both solution and the solid state, ruling out macroscopic anisotropic absorption artifacts (Figure S13).

The colloidal and chemical stability of ***L*‐TASe** nanoassemblies was evaluated over 30 days. DLS monitoring at 20 µM showed negligible changes in hydrodynamic diameter and size distribution, confirming excellent colloidal stability (Figure 3f). Incubation of preformed assemblies across pH 2.0–7.8 for 30 days, followed by ^1^H NMR analysis of the recovered solids, revealed no spectral changes, indicating intact molecular structure (Figure S14). Absorption analysis of the supernatants showed no detectable absorption from the precursor, excluding decomposition or leaching (Figure S15. These results collectively demonstrate the outstanding colloidal stability and pH‐tolerant chemical integrity of ***L*‐TASe** nanoassemblies.

To further examine the structural order of the aqueous assemblies, PXRD and small‐angle x‐ray scattering (SAXS) measurements were performed on ***L*‐TASe** nanoassemblies. The PXRD pattern of the freeze‐dried assemblies showed a broad diffuse feature centered at approximately 25°, without sharp Bragg reflections, indicating the absence of long‐range crystalline order (Figure S16a). Similarly, the one‐dimensional SAXS profile displayed a broad scattering feature, and the corresponding two‐dimensional pattern showed an isotropic scattering halo without discrete diffraction spots (Figure S16b,c). These results indicate the presence of short‐range intermolecular correlations but the absence of long‐range periodic order. Thus, the aqueous nanoassemblies are structurally distinct from the crystalline framework obtained by solid‐state crystallization, consistent with a noncrystalline or poorly crystalline character, as well as their uniform spherical morphology and colloidal stability in water.

Building on these observations, six structurally related control compounds, labelled as **TSe**, **TAPh**, **TMAPh**, **TPh**, **PASe**, and **TMASe** (Scheme 1d), were further examined by SEM, DLS, and CD spectroscopy to elucidate how the molecular structure influences the morphology and chiroptical properties of the aqueous assemblies. The control compounds exhibited diverse morphologies, including fibrous, sheet‐like, film‐like, spherical, and irregularly aggregated structures, with hydrodynamic diameters ranging from approximately 15 to 350 nm (Figures S17 and S18). Notably, **PASe** formed relatively ordered assemblies and displayed a strong CD signal, whereas the other control compounds showed only weak or negligible chiroptical responses. In particular, **TPh** and **TSe**, which lack amino acid moieties, exhibited no detectable CD signals (Figure S19). These comparative results suggest that the benzoselenadiazole unit, acylhydrazone/alanine motifs, and C_3_‐symmetric architecture collectively govern the morphology, size, and chiral expression of the TASe nanoassemblies.

### 2.5 Cardioprotective Performance and ROS‐Scavenging Activity

The stability of **TASe** nanoassemblies under cell culture conditions was first verified in Dulbecco's Modified Eagle Medium (DMEM) with or without 1% and 10% fetal bovine serum (FBS). Negligible changes in size distribution were observed, confirming that the nanoassembled state is preserved under cellular assay conditions (Figure S20).

**FIGURE 4:**
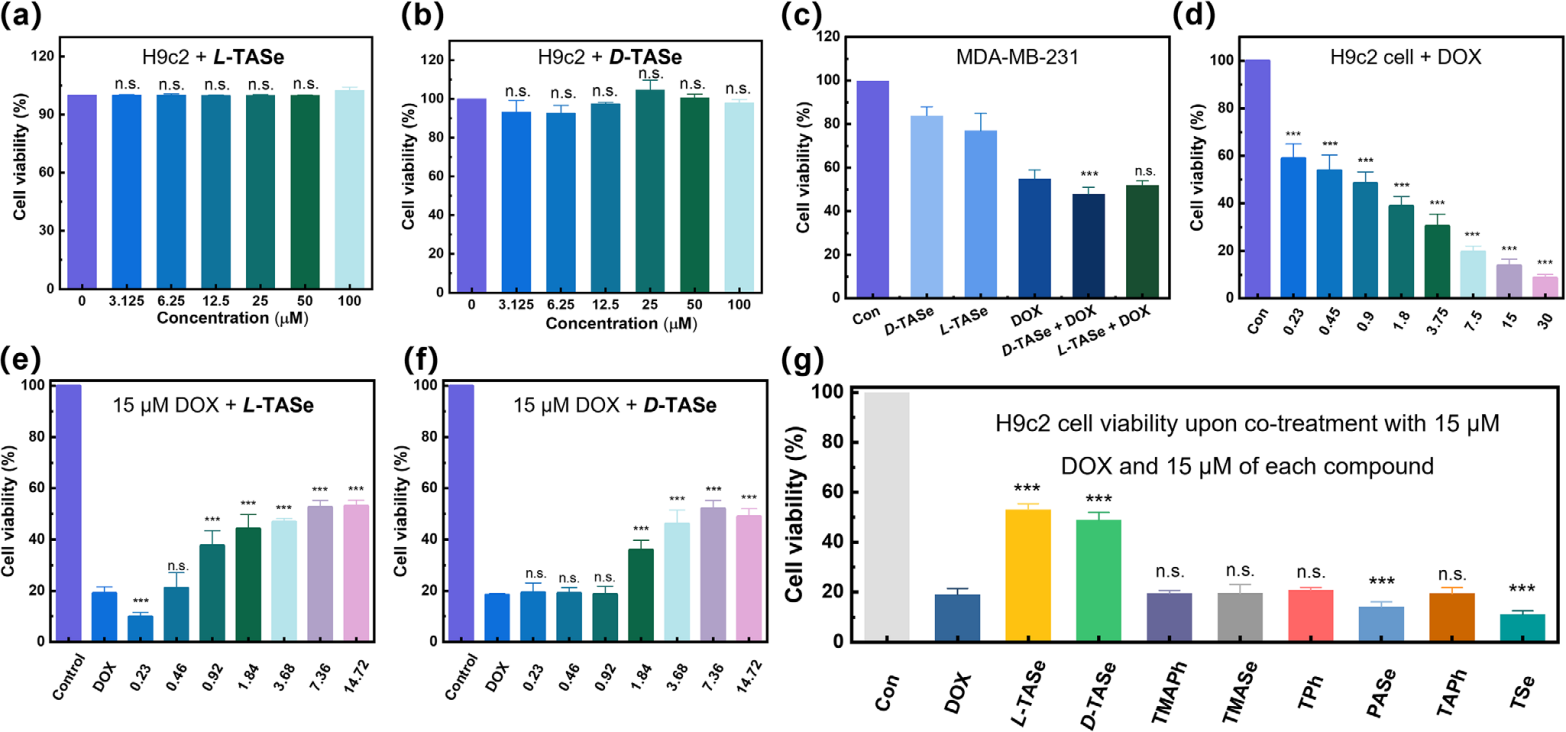
Biocompatibility and structure‐dependent cardioprotective performance of **TASe**. (a, b) Cell viability of H9c2 cardiomyocytes after 24 h treatment with increasing concentrations of ***L*‐TASe** and ***D*‐TASe**. (c) Cell viability of MDA‐MB‐231 breast cancer cells after treatment with DOX, ***L*‐TASe**, ***D*‐TASe**, or their combinations. (d) Dose‐response profile of H9c2 cells treated with DOX at indicated concentrations for 24 h. (e,f) Cardioprotection of ***L*‐TASe** and ***D***‐**TASe** against 15 µM DOX damage to H9c2 cardiomyocytes after 48 h treatment. (g) Comparative cytoprotective effects of 15 µM **TASe** and its structural analogs (**TMAPh**, **TMASe**, **TPh**, **PASe**, **TAPh**, **TSe**) against 15 µM DOX‐induced injury in H9c2 cardiomyocytes following 48 h incubation. Data are presented as mean ± SD from six independent experiments. Statistical significance was determined by one‐way ANOVA followed by Tukey's multiple‐comparisons test. n.s., not significant; ^*^ *P* < 0.05, ^**^ *P* < 0.01, ^***^ *P* < 0.001 versus DOX.

The cytocompatibility of **TASe** was assessed in H9c2 cardiomyocytes. Both ***L*‐TASe** and ***D*‐TASe** exhibited negligible cytotoxicity over the tested concentration range, with cell viability remaining comparable to untreated controls and no concentration‐dependent decline (Figure 4a,b). This excellent biocompatibility established a broad safety window for subsequent cardioprotection studies. To ensure that **TASe** does not compromise the anticancer efficacy of DOX, its effect on MDA‐MB‐231 breast cancer cells was examined. Free DOX markedly reduced cell viability, whereas co‐treatment with ***L*‐TASe** or ***D*‐TASe** did not rescue tumor cells from DOX‐induced inhibition. Instead, the **TASe** + DOX groups maintained comparable or slightly stronger inhibitory effects than DOX alone (Figure 4c). **TASe** alone also moderately reduced MDA‐MB‐231 cell viability, suggesting a possible auxiliary antitumor effect while retaining cardiomyocyte compatibility. The cardioprotective performance of **TASe** was evaluated in a DOX‐induced H9c2 injury model. Exposure to 15 µM DOX for 48 h dramatically decreased cell viability from ∼100% to ∼20%, establishing an acute severe injury model with a wide dynamic window for evaluating protective efficacy (Figure 4d). Co‐treatment with **TASe** substantially restored cell viability in a concentration‐dependent manner: ***L*‐TASe** increased viability from ∼20% to ∼53%, and ***D*‐TASe** to ∼52% at the highest tested concentration of 14.72 µM (Figure 4e,f). Both enantiomers exhibited comparable cardioprotective efficacy.

The protective effect was further validated under milder DOX conditions (1 and 5 µM), where **TASe** also produced appreciable cytoprotection (Figure S21). By contrast, a panel of structurally related control compounds failed to confer any appreciable protection under the same conditions (Figure 4g and Figures S22 and S23). These control compounds lack one or more of the key structural modules: compounds without the benzoselenadiazole unit (**TAPh**, **TMAPh**, **TPh**) or without the acylhydrazone/alanine framework (**TSe**, **TMASe**) all exhibited no protective activity. In addition, these control compounds exhibited diverse morphologies and sizes in aqueous solution, distinct from the uniform **TASe** nanospheres, further supporting that their altered assembly behavior contributes to the loss of cardioprotective activity (Figures S17–S19). Notably, the inactivity of **PASe**, which is derived from terephthalaldehyde instead of the 1,3,5‐benzenetricarboxaldehyde core of **TASe**, underscores the essential role of the C_3_‐symmetric phenyl core in enforcing the three‐armed geometry required for multivalent assembly and cooperative molecular interactions. Collectively, these results highlight that both the C_3_‐symmetric topology and the complete integration of the three functional modules are indispensable for the cardioprotective function.

To elucidate the mechanism underlying this cardioprotective effect, we first assessed whether **TASe** acts as a direct radical scavenger. 2,2‐Diphenyl‐1‐picrylhydrazyl (DPPH) assays showed that ***L*‐TASe** and ***D*‐TASe** exhibited only weak radical‐scavenging activity (<20% at most concentrations, ∼25% at 25 µM), whereas vitamin C achieved ∼80% scavenging at 30 µM (Figure S24). These results indicate that **TASe** does not act as a conventional small‐molecule antioxidant that primarily functions through direct radical quenching in solution via a stoichiometric mechanism. The DPPH assay probes a simple electron‐ or hydrogen‐transfer reaction in a homogeneous aqueous environment, which lacks the cofactors and subcellular environment required for the selenium‐centered processes that may operate within cells. Therefore, the weak DPPH activity does not preclude the ability of **TASe** to modulate intracellular oxidative stress through alternative pathways.

We next investigated whether the observed cardioprotection could instead arise from the sequestration of DOX by **TASe** assemblies. Free DOX displayed a characteristic absorption peak at approximately 484 nm, whereas ***L*‐TASe** showed negligible absorption in this spectral region (Figure S25). The ***L*‐TASe** + DOX mixture retained the characteristic DOX absorption at approximately 484 nm, with no appreciable alteration in its spectral features relative to free DOX. These results provide no evidence of substantial DOX encapsulation by ***L*‐TASe** assemblies under the tested conditions, indicating that the cardioprotective effect of ***L*‐TASe** is unlikely to result from the physical sequestration of DOX.

**FIGURE 5:**
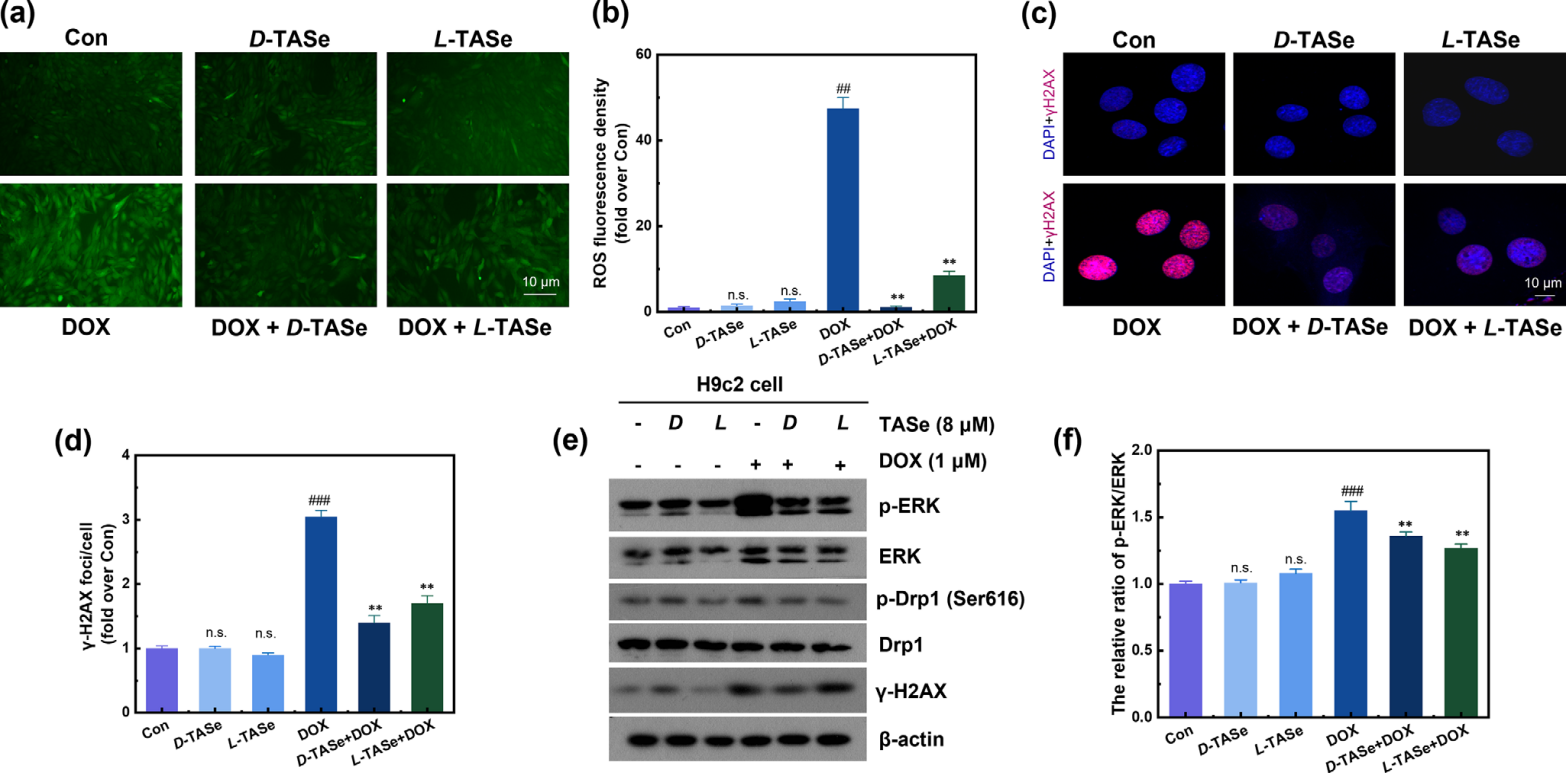
Mechanistic studies on the cardioprotective effects of ***L*‐TASe** and ***D*‐TASe** in H9c2 cells. (a) Representative DCFH‐DA fluorescence images showing intracellular ROS levels under different treatments. (b) Quantification of ROS fluorescence intensity, expressed as fold change relative to the control. (c) Representative immunofluorescence images of γ‐H2AX staining for DNA damage assessment. Nuclei were counterstained with DAPI. (d) Quantification of γ‐H2AX foci per cell, expressed as fold change relative to the control. (e) Western blot analysis of p‐ERK, ERK, and β‐actin expression. (f) Densitometric quantification of the p‐ERK/ERK ratio. Data are presented as mean ± SD from three independent experiments. Statistical significance was determined by one‐way ANOVA followed by Tukey's multiple‐comparisons test. n.s., not significant; ^*^ *P* < 0.05, ^**^ *P* < 0.01, ^***^ *P* < 0.001 versus DOX, ^##^ *P* ≤ 0.01, ^###^ *P* ≤ 0.001 versus the control.

Having ruled out both direct radical quenching and nanocarrier‐mediated DOX sequestration, we further examined whether **TASe** modulates intracellular ROS under DOX challenge using the DCFH‐DA probe. Untreated H9c2 cells and **TASe**‐alone groups displayed only weak fluorescence, whereas DOX exposure produced strong green fluorescence, confirming robust ROS generation. Co‐treatment with ***L*‐TASe** or ***D*‐TASe** markedly attenuated this signal, indicating effective suppression of DOX‐triggered ROS accumulation (Figure 5a,b). Interestingly, an opposite trend was observed in MDA‐MB‐231 cells, where **TASe** + DOX groups exhibited enhanced ROS fluorescence compared with DOX alone (Figure S26), suggesting a cell‐context‐dependent redox‐regulatory effect that alleviates oxidative stress in cardiomyocytes while enhancing it in tumor cells.

Excessive ROS accumulation is a key upstream event in DOX‐induced DNA damage. γ‐H2AX immunofluorescence staining revealed negligible signals in control and **TASe**‐alone groups, whereas DOX treatment produced pronounced nuclear foci. Co‐treatment with ***L*‐TASe** or ***D*‐TASe** markedly reduced γ‐H2AX accumulation, confirming attenuation of oxidative DNA damage (Figure 5c,d).

Western blot analysis (Figure 5e and Figure S27) showed that DOX markedly increased ERK phosphorylation without altering total ERK expression, indicating activation of the ERK pathway. Co‐treatment with **TASe** significantly reduced the p‐ERK/ERK ratio (Figure 5f), consistent with its ROS‐suppressing effect. DOX also disrupted mitochondrial dynamics, as evidenced by elevated p‐Drp1 (Ser616) expression (Figure S28), which was partially reversed by **TASe**.

Unlike conventional DOX nanocarriers that reduce cardiotoxicity primarily through altered biodistribution, **TASe** provides an intrinsically bioactive supramolecular platform with a chemically defined structure, stable nanoassembly, and traceable structure‐activity relationships (Table S3). Its selenium‐containing and acylhydrazone‐conjugated framework enables intracellular redox regulation, suppressing DOX‐induced ROS accumulation, oxidative DNA damage, and ERK activation in cardiomyocytes without compromising DOX's anticancer efficacy.

**FIGURE 6:**
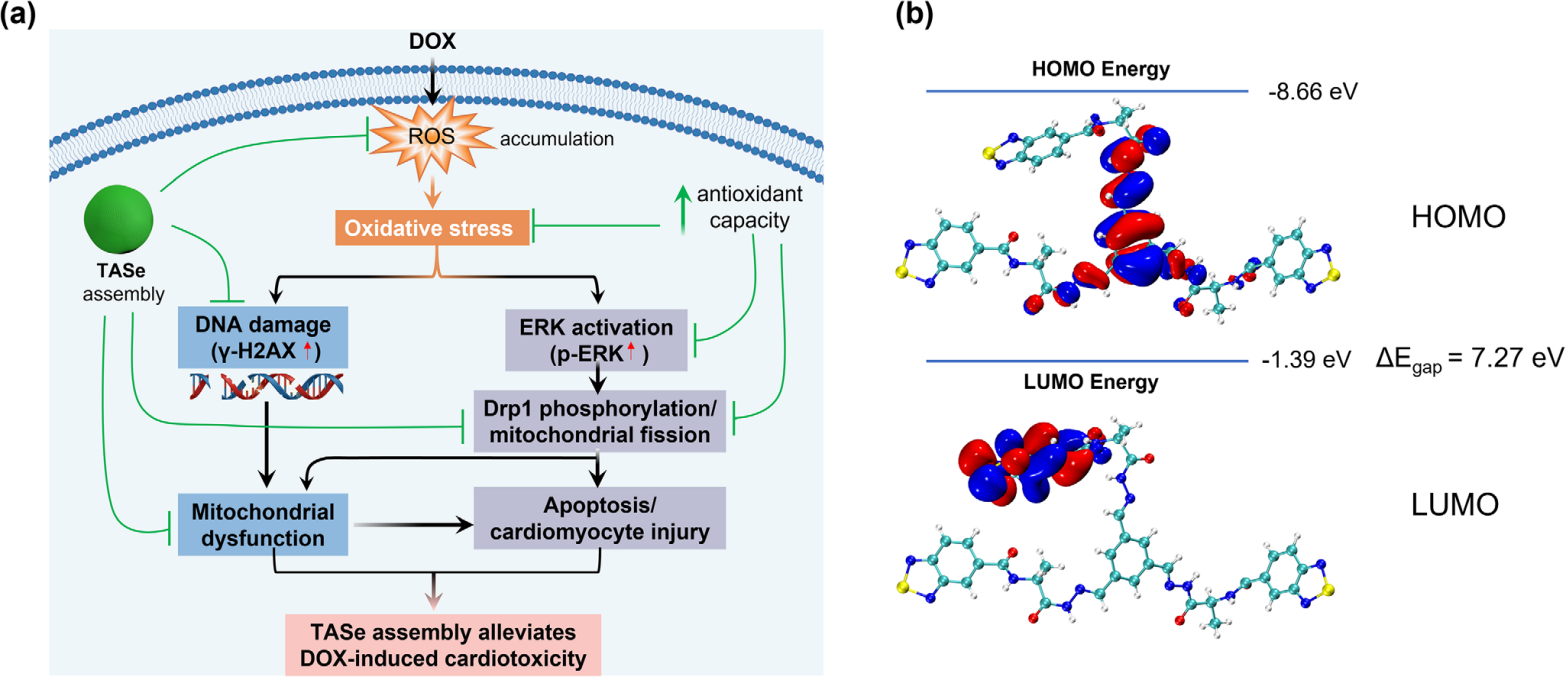
Proposed mechanism of **TASe**‐mediated cardioprotection and FMO analysis. (a) Schematic illustration of the cardioprotective mechanism of **TASe** assemblies, showing suppression of ROS accumulation and attenuation of downstream oxidative DNA damage, ERK activation, and mitochondrial stress. (b) FMO analysis of **TASe**. The HOMO (−8.66 eV) is mainly localized on the central aromatic/acylhydrazone region, whereas the LUMO (−1.39 eV) is delocalized over the benzoselenadiazole‐conjugated framework. The calculated HOMO‐LUMO gap is 7.27 eV.

These results demonstrate that **TASe** protects cardiomyocytes by interrupting the ROS‐DNA damage‐ERK signaling axis (Figure 6a). The weak DPPH activity contrasts with its potent intracellular ROS suppression, supporting a selenium‐associated redox regulatory mechanism involving intracellular redox modulation rather than direct radical quenching.

### 2.6 DFT Insights Into the Cardioprotective Mechanism

Density functional theory (DFT) calculations were performed at the B3LYP/6‐31G(d) level to elucidate the electronic basis of the antioxidant behavior of **TASe**. Molecular electrostatic potential (MEP) and frontier molecular orbital (FMO) analyses were conducted on the optimized structure to identify possible ROS‐interacting and electron‐transfer sites.

The MEP map revealed a heterogeneous electrostatic distribution on the **TASe** surface, with potential values ranging from ‐50.00 to 55.00 kcal mol^−1^ (Figure S29). Negative regions were mainly localized around the oxygen‐ and nitrogen‐containing acylhydrazone moieties, suggesting that these electron‐rich heteroatom sites may interact with electrophilic ROS or radical species. Positive regions were predominantly distributed around the peripheral hydrogen‐rich areas and terminal benzoselenadiazole units. This polarized surface indicates distinct electron‐rich and electron‐deficient regions that may facilitate ROS‐related redox interactions.

FMO analysis showed that the HOMO of **TASe** (−8.66 eV) is mainly localized on the central aromatic ring and adjacent acylhydrazone‐conjugated segments, identifying this region as the major electron‐donating site (Figure 6b). The LUMO (−1.39 eV) is distributed over the conjugated skeleton, including the selenium‐containing benzoselenadiazole unit. The calculated HOMO‐LUMO gap of 7.27 eV suggests a relatively stable electronic structure while retaining redox‐responsive regions for electron‐transfer processes.

These computational results establish the intrinsic electronic capacity of **TASe** to participate in redox interactions, which is consistent with the selenium‐centered redox chemistry known for organoselenium compounds [52, 53, 54]. The weak DPPH activity does not diminish this interpretation. The DPPH assay probes a stoichiometric electron‐ or hydrogen‐transfer reaction in a simple aqueous solution, whereas the intracellular environment presents a far more complex milieu. The redox‐active benzoselenadiazole units, when presented as part of the supramolecular nanoassemblies, may engage in cellular redox pathways through mechanisms that are not operational under cell‐free conditions.

Thus, the DFT analysis provides a molecular‐level rationale for the redox‐modulating potential of the **TASe** building block, while the cellular experiments demonstrate that this potential is expressed specifically within the intracellular environment through the cooperative action of the supramolecular assemblies.

## 3 Conclusion

In summary, we have developed **TASe**, a C_3_‐symmetric supramolecular building block that covalently integrates benzoselenadiazole, alanine, and acylhydrazone modules within a single scaffold. Cooperative hydrogen and chalcogen bonds drive the formation of a crystalline framework with permanent porosity in the solid state, while in aqueous media the same molecular modules promote self‐assembly into uniform chiral nanospheres. The resulting assemblies protect cardiomyocytes from DOX‐induced oxidative injury by suppressing ROS accumulation, mitigating DNA damage, and attenuating ERK activation, without compromising the anticancer activity of the drug. By embedding both assembly‐directing and bioactive functions within a single molecular platform, this work establishes a generalizable design paradigm for constructing supramolecular materials with unified structural order and therapeutic function, exemplifying the aggregatism paradigm, where emergent functions arise from supramolecular assemblies beyond the capabilities of isolated molecules.

## 4 Experimental Methods

### 4.1 Materials

All chemicals, solvents, and biological reagents were purchased from commercial suppliers and used as received unless otherwise stated.

Doxorubicin hydrochloride (DOX), 2,2‐diphenyl‐1‐picrylhydrazyl (DPPH), vitamin C (VC), 2’,7’‐dichlorodihydrofluorescein diacetate (DCFH‐DA), 4’,6‐diamidino‐2‐phenylindole (DAPI), phosphate‐buffered saline (PBS), dimethyl sulfoxide (DMSO), paraformaldehyde, Triton X‐100, bovine serum albumin (BSA), RIPA lysis buffer, protease/phosphatase inhibitor cocktail, Dulbecco's modified Eagle's medium (DMEM), fetal bovine serum (FBS), penicillin‐streptomycin, trypsin‐EDTA, Cell Counting Kit‐8 (CCK‐8), and cell culture consumables were obtained from standard commercial sources. Primary antibodies against p‐ERK1/2, ERK1/2, γ‐H2AX, p‐Drp1 (Ser616), Drp1, and β‐actin, together with the corresponding horseradish peroxidase (HRP)‐conjugated or fluorescence‐labeled secondary antibodies, were purchased commercially and used according to the manufacturers’ instructions.

### 4.2 General Characterization

Single‐crystal x‐ray diffraction data were collected on an Agilent SuperNova Dual diffractometer at 100 K using Cu Kα radiation, λ = 1.54184 Å, or Mo Kα radiation, λ = 0.71073 Å. The crystal structures were solved by direct methods using the OLEX2 software package. Unless otherwise specified, all non‐hydrogen atoms were refined anisotropically. Absorption corrections were applied using the multiscan method implemented in the CrysAlis software package. Powder x‐ray diffraction (PXRD) patterns were recorded on a SmartLab diffractometer, Rigaku, Japan, using Cu Kα radiation at room temperature with a step size of 0.02°. Density functional theory (DFT) calculations were performed using Gaussian 16, and electrostatic potential maps were analyzed using Multiwfn. Fourier‐transform infrared (FTIR) spectra were recorded on a Nicolet AVATAR FT‐IR330 spectrometer. Circular dichroism (CD) spectra were measured using a JASCO J‐1700 spectrometer. Scanning electron microscopy (SEM) images were obtained using a ThermoFisher Helios 5 UC microscope. ^1^H and ^13^C NMR spectra were recorded on a Bruker AV600 MHz NMR spectrometer in DMSO‐*d*_6_. High‐resolution mass spectrometry (HRMS) data were acquired using a Thermo Q Exactive LC‐MS/MS system. Nitrogen adsorption measurements were performed using an ASAP 2020 Plus Version 2.00 automatic surface area and porosity analyzer, Micromeritics, USA. Static water vapor adsorption measurements were conducted using a BSD‐660MV instrument, Beishide Instrument. Before gas adsorption measurements, samples were evacuated at 100°C for 2 h, and adsorption isotherms were collected at 298 K. Thermogravimetric analysis (TGA) was performed on an SDT 650‐Discovery MS simultaneous DSC/TGA‐mass spectrometry system from 30°C to 800°C under an N_2_ atmosphere. Small‐angle x‐ray scattering (SAXS) measurements were performed on a Xeuss 3.0 HR small‐angle x‐ray scattering system (Xenocs) using Cu Kα radiation (λ = 1.5418 Å) at room temperature.

Additional characterization data for the synthesized compounds, including ^1^H NMR, ^13^C NMR, and HRMS spectra, are provided in Figures S30–S52.

### 4.3 Preparation of Stock Solutions and TASe Nanoassemblies

***L*‐TASe**, ***D*‐TASe**, and structurally related control compounds, including **TSe**, **TAPh**, **TMAPh**, **TPh**, **PASe**, and **TMASe**, were dissolved in DMSO to prepare stock solutions. Before use, the stock solutions were freshly diluted with water or culture medium. For all cell‐based experiments, the final DMSO concentration was maintained below 0.1% v/v, and vehicle control groups received an equivalent amount of DMSO. For self‐assembly experiments, concentrated DMSO stock solutions of ***L*‐TASe** or ***D*‐TASe** were slowly diluted into deionized water or culture medium under gentle vortexing or sonication to obtain homogeneous dispersions. The resulting dispersions were equilibrated at room temperature before characterization. For biological experiments, freshly prepared **TASe** nanoassemblies were further diluted with complete culture medium to the desired concentrations immediately before use.

### 4.4 Variable‐Temperature ^1^H NMR Spectroscopy

Variable‐temperature ^1^H NMR spectra of ***L*‐TASe** in DMSO‐*d*_6_ were recorded on a Bruker AV600 MHz NMR spectrometer over the temperature range of 25°C–100°C. The sample was allowed to equilibrate at each temperature before data acquisition. Chemical shifts were referenced to the residual solvent signal of DMSO‐*d*_6_.

### 4.5 Fluorescence Spectroscopy

Concentration‐dependent fluorescence spectra of aqueous ***L*‐TASe** dispersions were recorded at room temperature. Samples were excited at approximately 350 nm, and emission spectra were collected over the appropriate wavelength range. The emission intensity at 705 nm was used to evaluate the concentration‐dependent photoluminescence behavior of ***L*‐TASe**.

### 4.6 Dynamic Light Scattering

Dynamic light scattering (DLS) was used to determine the hydrodynamic diameter and size distribution of **TASe** assemblies. All measurements were performed at room temperature. Concentration‐dependent DLS measurements were conducted over a **TASe** concentration range of 5–40 µM. For long‐term colloidal stability evaluation, ***L***‐**TASe** assemblies, 20 µM, were incubated under ambient conditions for 30 days and analyzed by DLS at predetermined time points. To assess their stability in biological media, **TASe** assemblies were incubated in DMEM, DMEM supplemented with 1% FBS, or DMEM supplemented with 10% FBS, followed by DLS analysis.

### 4.7 SEM and TEM Characterization

For SEM measurements, **TASe** dispersions were deposited onto clean silicon wafers and dried under ambient conditions. SEM images were acquired using a ThermoFisher Helios 5 UC microscope, and particle size distributions were analyzed using Nano Measurer software. For TEM measurements, a drop of **TASe** dispersion was deposited onto a carbon‐coated copper grid. After standing for a short period, excess solution was removed with filter paper, and the grid was dried before imaging.

### 4.8 CD and Absorption Spectroscopy

Aqueous dispersions of ***L*‐TASe** and ***D*‐TASe** were prepared under identical conditions for CD and absorption measurements. CD spectra were recorded on a JASCO J‐1700 spectrometer at room temperature using quartz cuvettes. Absorption spectra were acquired using an Agilent Cary 60 spectrophotometer.

### 4.9 pH Stability Assay

Preformed ***L*‐TASe** assemblies were incubated in aqueous media at pH 2.0, 4.0, 5.0, 7.0, and 7.8 for 30 days. After incubation, the samples were centrifuged. The recovered solids were dried and redissolved in DMSO‐*d*_6_ for ^1^H NMR analysis. The corresponding supernatants were analyzed by absorption spectroscopy.

### 4.10 Cell Culture

Rat embryonic cardiomyoblast H9c2 cells and human triple‐negative breast cancer MDA‐MB‐231 cells were purchased from Procell Life Science & Technology Co., Ltd. (Wuhan, China). The cells were cultured in high‐glucose DMEM supplemented with 10% fetal bovine serum and 1% penicillin‐streptomycin and maintained at 37 °C in a humidified incubator with 5% CO_2_. The culture medium was replaced every 2 days, and the cells were passaged with trypsin‐EDTA when they reached 80–90% confluence. Cells in the logarithmic growth phase were used for all experiments.

### 4.11 Cell Viability Assay

Cell viability was determined using the CCK‐8 assay. H9c2 or MDA‐MB‐231 cells were seeded into 96‐well plates and allowed to adhere overnight. After the indicated treatments, CCK‐8 solution was added according to the manufacturer's instructions, and the cells were incubated at 37°C. The absorbance at 450 nm was then measured using a microplate reader. Cell viability was calculated by normalizing the absorbance of each treatment group to that of the untreated control group. For cytocompatibility assays, H9c2 cells were treated with different concentrations of ***L***‐**TASe** or ***D***‐**TASe**. For cardioprotection assays, H9c2 cells were treated with DOX in the absence or presence of **TASe** or control compounds. For tumor‐cell assays, MDA‐MB‐231 cells were treated with DOX, **TASe**, or their combinations.

### 4.12 DOX‐Induced H9c2 Cell Injury Model

H9c2 cells were seeded and allowed to adhere overnight. For the acute injury model, cells were exposed to 15 µM DOX. For protection experiments, cells were treated with ***L*‐TASe** or ***D*‐TASe** in the presence of DOX. Unless otherwise specified, 15 µM **TASe** was used in the main protection experiments under 15 µM DOX challenge. Untreated cells, **TASe**‐treated cells, DOX‐treated cells, and **TASe** + DOX‐treated cells were included as experimental groups. For mild injury models, cells were treated with 1 or 5 µM DOX in the absence or presence of **TASe**.

### 4.13 Evaluation of DOX Antitumor Activity

MDA‐MB‐231 cells were seeded into 96‐well plates and treated with DOX, ***L*‐TASe**, ***D*‐TASe**, ***L*‐TASe** + DOX, or ***D*‐TASe** + DOX at the indicated concentrations. After incubation, cell viability was determined using the CCK‐8 assay.

### 4.14 Structure‐Activity Relationship Comparison

To evaluate the structure dependence of the cardioprotective effect, H9c2 cells were co‐treated with DOX at 15, 5, and 1 µM and the corresponding concentrations of **TSe**, **TAPh**, **TMAPh**, **TPh**, **PASe**, **TMASe**, ***L*‐TASe**, and ***D*‐TASe**. Cell viability was subsequently measured using the CCK‐8 assay.

### 4.15 DPPH Radical‐Scavenging Assay

Freshly prepared DPPH solution was mixed with different concentrations of ***L*‐TASe**, ***D*‐TASe**, and VC, with final compound concentrations ranging from 1 to 40 µM. After incubation in the dark at room temperature, the absorbance of DPPH at 517 nm was measured using a microplate reader. The radical‐scavenging efficiency was calculated using the following equation:

$$\mathrm{Scavenging}\;\mathrm{efficiency}\;\left(\%\right)=1-\left(\frac{A_{sample}}{A_{control}}\right)\times 100\%$$

where *A*_sample_ and *A*_control_ represent the absorbance values of DPPH solutions with and without the tested compound, respectively.

### 4.16 Intracellular ROS Detection

Intracellular ROS levels were detected using DCFH‐DA staining. After the indicated treatments, H9c2 or MDA‐MB‐231 cells were washed with PBS and incubated with DCFH‐DA working solution in serum‐free DMEM at 37°C for 20–30 min in the dark. The cells were then washed three times with PBS and imaged using a fluorescence microscope under identical imaging settings. Fluorescence intensity was quantified using ImageJ software.

### 4.17 γ‐H2AX Immunofluorescence Staining

H9c2 cells grown on coverslips were subjected to the indicated treatments, fixed with 4% paraformaldehyde for 15 min, permeabilized with 0.1% Triton X‐100, and blocked with 5% BSA. The cells were incubated with an anti‐γ‐H2AX primary antibody overnight at 4°C, followed by incubation with a fluorescence‐labeled secondary antibody for 1 h at room temperature in the dark. Nuclei were counterstained with DAPI. Images were acquired using a fluorescence microscope, and γ‐H2AX fluorescence intensity was quantified using ImageJ software.

### 4.18 Western Blot Analysis

After treatment, H9c2 cells were lysed in RIPA lysis buffer supplemented with protease and phosphatase inhibitors. Cell lysates were centrifuged at 12,000 rpm for 15 min at 4°C, and protein concentrations were determined using the BCA assay. Equal amounts of protein were separated by SDS‐PAGE and transferred onto PVDF membranes. The membranes were blocked with 5% non‐fat milk or BSA and incubated overnight at 4°C with primary antibodies against p‐ERK1/2, ERK1/2, p‐Drp1 (Ser616), Drp1, γ‐H2AX, β‐actin. After washing, the membranes were incubated with HRP‐conjugated secondary antibodies. Protein bands were visualized using enhanced chemiluminescence. Band intensities were quantified using ImageJ software. p‐ERK1/2 levels were normalized to total ERK1/2, whereas other protein levels were normalized to β‐actin.

### 4.19 Statistical Analysis

Biological data were analyzed using Origin Software. Data are presented as mean ± SD from six independent experiments (n = 6), unless otherwise indicated. Cell viability, intracellular ROS, γ‐H2AX fluorescence, DPPH‐scavenging activity, and western blot data were normalized to the corresponding control or loading‐control group before analysis. Technical replicates were averaged and were not treated as independent biological replicates. Data were excluded only when a documented technical error occurred. Statistical significance was assessed using one‐way ANOVA followed by Tukey's multiple‐comparisons test. *P* < 0.05 was considered statistically significant. The exact sample size and statistical test are indicated in the corresponding figure legends.

## Author Contributions

Jinkui Teng, Renjing Yang, and Yu Du contributed equally to this work. Xiaosheng Yan, Yun‐Bao Jiang, Haifeng Chen, and Aijie Liu conceived and supervised the project. Jinkui Teng designed the molecular synthesis, performed the material preparation and characterization. Renjing Yang, Yu Du, Guanghui Wang, Wenjing Tian, Yiming Xu, and Zhihua He performed the cellular experiments. Jinkui Teng, Renjing Yang, Yu Du, and Jingzhong Duan conducted the data analysis and prepared the figures. Jinkui Teng wrote the original draft. Aijie Liu, Haifeng Chen, Yun‐Bao Jiang, and Xiaosheng Yan revised and edited the manuscript. All authors discussed the results and approved the final version of the manuscript.

## Conflicts of Interest

The authors declare no conflicts of interest.

## Supporting information




**Supporting File**: advs77953‐sup‐0001‐SuppMat.doc.

## Data Availability

The data that support the findings of this study are available from the corresponding author upon reasonable request.
