# Supplementary material for: Supramolecular Chiral Assemblies from Benzoselenadiazole‐Alanine‐Acylhydrazone Conjugates Enable Cardioprotection
Source: Adv Sci (Weinh). 2026 Sep 27:e77953. Online ahead of print. doi: 10.1002/advs.77953 (PMC13616315; doi:10.1002/advs.77953)
Supplement: Supplementary file 1 [file ADVS-9999-e77953-s001.doc]

**Supramolecular Chiral Assemblies from Benzoselenadiazole-Alanine-Acylhydrazone Conjugates Enable Cardioprotection**

Jinkui Teng,^[a+]^ Renjing Yang,^[a+]^ Yu Du,^[a+]^ Jingzhong Duan,^[a]^ Yiming Xu,^[c]^ Zhihua He,^[c]^ Aijie Liu,*^[ad]^ Haifeng Chen,*^[a]^ Yun-Bao Jiang,*^[b]^ and Xiaosheng Yan*^[abd]^

*^a^* Fujian Provincial Key Laboratory of Innovative Drug Target Research, State Key Laboratory of Vaccines for Infectious Diseases, Xiang An Biomedicine Laboratory, School of Pharmaceutical Sciences, Xiamen University, Xiamen, Fujian 361102, China.

*^b^* Department of Chemistry, College of Chemistry and Chemical Engineering, The MOE Key Laboratory of Spectrochemical Analysis and Instrumentation, Xiamen University, Xiamen, Fujian 361005, China.

*^c^* Zhongshan Hospital Xiamen University, Xiamen, Fujian 361004, China.

*^d^* Jiujiang Research Institute of Xiamen University, Jiujiang, Jiangxi 332000, China.

^+^ These authors contributed equally to this work.

*E-mail: aijieliu@xmu.edu.cn, haifeng@xmu.edu.cn, ybjiang@xmu.edu.cn, xshyan@xmu.edu.cn

**Supporting Information**

**Contents**

**1. Syntheses and characterizations	S2**

**2. Structural and crystal analysis	S10**

**3. Solid-state characterization of chiral TASe assembly	S14**

**4. Solution-phase assembly	S15**

**5. Antioxidative, cardioprotective and cytotoxic activities	S20**

**6. ^1^H NMR, ^13^C NMR and HRMS spectra	S33**

**7. References	S45**

1. Syntheses and characterizations

**Scheme S1**. General procedures for the syntheses of **TASe**.

*R-1/S-1*

**1d:** The synthesis of **1d**, referring to our previous work.^[1]^

**TASe:** A mixture of compound **1d** (0.97 g, 3.1 mmol) dissolved in 40 mL of deionized water and 1,3,5-benzenetricarboxaldehyde (**TFB**) (0.162 g, 1 mmol) dissolved in 20 mL of ethanol was prepared in a 100 mL round-bottom flask. The combined solution was stirred at ambient temperature for 30 minutes, during which a white precipitate formed. The reaction mixture was subsequently heated to 70ºC. The precipitate was isolated by hot filtration, washed with cold ethanol (3 × 5 mL), and dried under vacuum to afford a white solid (0.90 g, 90% yield).

***L*-TASe**: Mp = 247.4~248.8 ºC. ^1^H NMR (600 MHz, DMSO-*d*_6_) δ 11.81 – 11.73 (d, *J* = 6.3 Hz, 3H), 11.65 – 11.56 (t, *J* = 8.3 Hz, 3H), 9.10 – 8.95 (m, 6H), 8.52 – 8.34 (m, 9H), 8.22 – 7.83 (m, 21H), 5.47 – 5.38 (dh, *J* = 14.9, 7.2 Hz, 3H), 4.63 – 4.56 (p, *J* = 7.1 Hz, 3H), 1.53 – 1.45 (ddd, *J* = 17.9, 7.3, 5.1 Hz, 18H). ^13^C NMR (151 MHz, DMSO-*d*_6_) δ 174.44, 174.35, 174.21, 169.60, 166.11, 165.84, 165.79, 165.75, 160.79, 160.77, 160.73, 159.72, 159.70, 146.50, 146.40, 146.27, 142.85, 142.47, 142.25, 136.09, 136.02, 135.97, 135.91, 135.81, 134.57, 134.45, 128.40, 128.37, 128.35, 126.98, 126.85, 126.07, 125.43, 123.34, 123.28, 123.26, 123.19, 123.00, 122.95, 122.93, 49.26, 49.22, 49.19, 47.05, 46.99, 46.90, 18.02, 17.09, 17.03. HRMS (ESI): calcd for [C_39_H_34_N_15_O_6_Se_3_]+:1048.0312, found: 1048.0327.

***D*-TASe**: Mp = 248.4~250.2 ºC. ^1^H NMR (600 MHz, DMSO-*d*_6_) δ 11.81 – 11.73 (d, *J* = 6.3 Hz, 3H), 11.65 – 11.56 (t, *J* = 8.3 Hz, 3H), 9.10 – 8.95 (m, 6H), 8.52 – 8.34 (m, 9H), 8.22 – 7.83 (m, 21H), 5.47 – 5.38 (dh, *J* = 14.9, 7.2 Hz, 3H), 4.63 – 4.56 (p, *J* = 7.1 Hz, 3H), 1.53 – 1.45 (ddd, *J* = 17.9, 7.3, 5.1 Hz, 18H). ^13^C NMR (151 MHz, DMSO-*d*_6_) δ 174.43, 174.35, 174.21, 169.60, 166.11, 165.84, 165.79, 165.75, 160.79, 160.77, 160.73, 159.72, 159.70, 146.49, 146.39, 146.26, 142.85, 142.46, 142.24, 136.09, 136.02, 135.98, 135.91, 135.82, 134.58, 134.45, 128.40, 128.38, 128.35, 126.98, 126.92, 126.85, 126.08, 123.34, 123.27, 123.19, 123.00, 122.95, 122.93, 49.26, 49.22, 49.18, 47.04, 46.99, 46.90, 18.02, 17.09, 17.03, 17.01. HRMS (ESI): calcd for [C_39_H_34_N_15_O_6_Se_3_]+:1048.0312, found: 1048.0328.

**Scheme S2**. General procedures for the syntheses of **PASe**.

**PASe:** Compound **1d** (0.66 g, 2.1 mmol) was dissolved in water (40 mL) in a 100 mL round-bottom flask. Separately, p-phthalaldehyde (**1,4-PDA**) (0.134 g, 1.0 mmol) was dissolved in absolute ethanol (10 mL). The two solutions were combined and stirred at room temperature for 30 minutes, resulting in the formation of a white precipitate. The suspension was then heated to 70 ºC and maintained at this temperature for 2 minutes. The solid was collected by hot filtration, washed with cold ethanol (20 mL), and dried under vacuum to afford the product as a white solid (0.65 g, 90% yield).

**PASe**: Mp = 337.8~339.5 ºC. ^1^H NMR (600 MHz, DMSO-*d*_6_) δ 11.74 – 11.68 (d, *J* = 5.5 Hz, 2H), 11.58 – 11.49 (d, *J* = 6.9 Hz, 2H), 9.11 – 9.05 (d, *J* = 6.9 Hz, 2H), 9.05 – 8.99 (d, *J* = 7.3 Hz, 2H), 8.53 – 8.46 (d, *J* = 10.7 Hz, 4H), 8.32 – 8.26 (d, *J* = 6.3 Hz, 2H), 8.07 – 8.04 (s, 2H), 8.01 – 7.95 (d, *J* = 9.2 Hz, 4H), 7.95 – 7.89 (d, *J* = 9.3 Hz, 4H), 7.80 – 7.74 (s, 7H), 5.41 – 5.34 (p, *J* = 7.3 Hz, 2H), 4.61 – 4.53 (p, *J* = 7.3 Hz, 2H), 1.52 – 1.44 (m, 12H). ^13^C NMR (151 MHz, DMSO-*d*_6_) δ 174.19, 169.47, 166.09, 165.84, 160.80, 159.74, 146.79, 146.69, 143.19, 143.14, 136.13, 136.02, 135.98, 135.89, 134.65, 134.46, 128.39, 127.93, 127.87, 127.66, 127.62, 123.38, 123.35, 123.18, 122.98, 49.19, 46.95, 18.03, 17.07. HRMS (ESI): calcd for [C_28_H_25_N_10_O_4_Se_2_]^+^: 725.0391, found: 725.0414.

**Scheme S3**. General procedures for the syntheses of **TAPh**.

**2c:** To a solution of benzoyl chloride (**2a**) (1.4 g, 10.0 mmol) and ethyl L-alaninate hydrochloride (**L-AlaOEtHCl**) (1.54 g, 10.1 mmol) in dichloromethane (DCM, 100 mL) was added triethylamine (TEA, 3.2 mL, 20.0 mmol). The reaction mixture was stirred at room temperature for 4 hours. Upon completion, approximately 40 mL of water was added to the round-bottom flask, and the mixture was transferred to a separatory funnel. The organic phase was sequentially washed with dilute aqueous ammonia (1%), hydrochloric acid (1%), and saturated aqueous sodium chloride. The lower organic layer was collected, and the solvent was evaporated under reduced pressure to afford **2b** as a white solid (1.7 g, 80% yield).

To a solution of the above white solid (1.7 g, 8 mmol) in ethanol (EtOH, 100 mL) was added hydrazine hydrate (10 mL, 40.0 mmol). The mixture was refluxed and stirred for 24 hours, during which a large amount of white precipitate formed. The precipitate was collected by filtration, washed sequentially with acetonitrile and ethanol, and dried to give **2c** as a white solid (1.24 g, 70% yield).

**TAPh:** Compound **2c** (0.64 g, 3.1 mmol) was dissolved in water (40 mL) in a 100 mL round-bottom flask. Separately, **TFB** (0.162 g, 1 mmol) was dissolved in absolute ethanol (10 mL). The two solutions were combined and stirred at room temperature for 30 minutes, resulting in the formation of a white precipitate. The suspension was then heated to 70 ºC and maintained at this temperature for 2 minutes. The solid was collected by hot filtration, washed with cold ethanol (20 mL), and dried under vacuum to afford the product as a white solid (0.66g, 90% yield).

**TAPh**: Mp = 254.5~259.7 ºC. ^1^H NMR (600 MHz, DMSO-*d*_6_) δ 11.75 – 11.65 (d, *J* = 8.4 Hz, 3H), 11.57 – 11.49 (t, *J* = 5.0 Hz, 3H), 8.79 – 8.54 (m, 6H), 8.46 – 8.28 (m, 3H), 8.20 – 7.86 (m, 18H), 7.62 – 7.37 (m, 15H), 5.47 – 5.29 (m, 3H), 4.60 – 4.49 (p, *J* = 7.1 Hz, 3H), 1.49 – 1.40 (ddd, *J* = 16.2, 7.2, 2.3 Hz, 18H). ^13^C NMR (151 MHz, DMSO-*d*_6_) δ 174.49, 174.41, 169.80, 166.80, 166.52, 146.23, 146.10, 142.72, 142.45, 142.25, 136.02, 135.94, 135.89, 135.82, 134.50, 134.33, 131.84, 131.73, 128.66, 128.64, 128.06, 127.97, 126.96, 126.86, 126.75, 126.03, 48.92, 46.71, 46.63, 18.06, 17.18, 17.06, 17.00. HRMS (ESI): calcd for [C_39_H_40_N_9_O_6_]^+^: 730.3102, found: 730.3106.

**Scheme S4**. General procedures for the syntheses of **TMASe**.

**TMASe:** Trimesoyl chloride (**TMC**, 0.26 g, 1.0 mmol) and compound **1d** (0.97 g, 3.1 mmol) were dissolved in anhydrous N, N-dimethylformamide (DMF, 20 mL). To this solution, triethylamine (TEA, 500 μL, 3.6 mmol) was added dropwise. The resulting reaction mixture was stirred continuously at 25 ºC for 4 h. Upon completion, the DMF solvent was removed under reduced pressure using rotary evaporation. Deionized water (20 mL) was added to the residue, and the mixture was dispersed by ultrasonication. The aqueous suspension was heated to 75 ºC and maintained at this temperature for 2 min. Hot filtration was immediately performed, and the solid product was collected. The crude product was transferred to a vacuum drying oven and dried at70ºC for 2 h to afford the target compound **TMASe** as a yellow solid (0.93 g, 85% yield).

**TMASe**: Mp = 250.6~257.3 ºC. ^1^H NMR (600 MHz, DMSO-*d*_6_) δ 10.91 – 10.68 (d, *J* = 55.3 Hz, 3H), 10.38 – 10.24 (d, *J* = 14.9 Hz, 3H), 9.11 – 8.96 (d, *J* = 7.2 Hz, 3H), 8.67 – 8.58 (s, 3H), 8.54 – 8.45 (s, 3H), 8.04 – 7.86 (m, 7H), 4.74 – 4.62 (p, *J* = 7.2 Hz, 3H), 1.58 – 1.42 (d, *J* = 7.1 Hz, 9H). ^13^C NMR (151 MHz, DMSO-*d*_6_) δ 172.22, 166.01, 164.86, 160.78, 159.72, 134.61, 133.80, 131.61, 128.48, 123.30, 123.21, 48.53, 18.44. HRMS (ESI): calcd for [C_39_H_34_N_15_O_9_Se_3_]^+^: 1096.0160, found: 1096.0249.

**Scheme S5**. General procedures for the syntheses of **TMAPh**.

**TMAPh:** Trimethylbenzoyl chloride (**TMC**, 0.26 g, 1.0 mmol) and compound **2c** (0.64 g, 3.1 mmol) were dissolved in *N, N*-dimethylformamide (DMF, 20 mL). Triethylamine (TEA, 500 μL, 3.6 mmol) was then added to the solution. The resulting reaction mixture was stirred continuously at room temperature (25 ºC) for 4 hours. Upon completion of the reaction, the DMF solvent was removed by rotary evaporation under reduced pressure. Deionized water (20 mL) was added to the residue, and after dispersion via ultrasonication, the mixture was heated to 75 ºC and maintained for 2 minutes. Hot filtration was subsequently performed to collect the solid product. This solid was transferred to a vacuum drying oven and dried to afford the target product **TMAPh** as a yellow solid (0.54 g), yielding 70%.

**TMAPh**: Mp = 214.5~216.8 ºC. ^1^H NMR (600 MHz, DMSO-*d*_6_) δ 10.94 – 10.54 (d, *J* = 81.3 Hz, 3H), 10.43 – 10.10 (d, *J* = 15.9 Hz, 3H), 8.70 – 8.64 (d, *J* = 7.4 Hz, 3H), 8.63 – 8.48 (d, *J* = 47.5 Hz, 4H), 7.96 – 7.90 (d, *J* = 7.7 Hz, 6H), 7.58 – 7.51 (t, *J* = 7.4 Hz, 3H), 7.51 – 7.44 (t, *J* = 7.6 Hz, 6H), 4.68 – 4.61 (p, *J* = 7.3 Hz, 3H), 1.63 – 1.37 (d, *J* = 7.2 Hz, 9H). ^13^C NMR (151 MHz, DMSO-*d*_6_) δ 172.39, 166.64, 166.63, 164.82, 164.76, 134.48, 133.74, 131.79, 131.54, 130.08, 128.63, 128.07, 48.21, 48.18, 18.51. HRMS (ESI): calcd for [C_39_H_40_N_9_O_9_]^+^: 778.2949, found: 778.2971.

**Scheme S6**. General procedures for the syntheses of **TPh**.

**3b:** To a solution of methyl benzoate (**3a**) (2.7 g, 20.0 mmol) in ethanol (EtOH, 100 mL), hydrazine hydrate (N_2_H_4_·H_2_O, 80%, 10 mL, 160.0 mmol) was added. The reaction mixture was stirred under reflux overnight. After completion, it was cooled to room temperature and filtered. The solid was washed with ethanol (30 mL) to afford the product (**3b**) as a white solid (1.4 g, 56% yield).

**TPh:** To a solution of **3b** (0.42 g, 3.1 mmol) and **TFB** (0.162 g, 1 mmol) in water (50 mL), the reaction mixture was stirred at room temperature for 1 hour. Upon completion, the precipitate was collected by filtration and washed sequentially with water (50 mL) and diethyl ether (15 mL). The solid was dried in a vacuum oven for 24 hours to afford the target compound **TPh** as a white solid (0.49 g, 95% yield).

**TPh**: Mp = 381.3~385.4 ºC. ^1^H NMR (600 MHz, DMSO-*d*_6_) δ 12.18 – 11.94 (d, *J* = 8.2 Hz, 3H), 8.66 – 8.52 (d, *J* = 13.4 Hz, 3H), 8.36 – 8.10 (d, *J* = 119.6 Hz,3H), 8.01 – 7.89 (m, 3H), 7.67 – 7.52 (m, 12H). ^13^C NMR (151 MHz, DMSO-*d*_6_) δ 163.85, 147.04, 136.10, 133.78, 132.39, 129.03, 128.19, 127.07. HRMS (ESI): calcd for [C_30_H_25_N_6_O_3_]^+^: 517.1988, found: 517.2002.

**Scheme S7**. General procedures for the syntheses of **TSe**.

**4a**: To a suspension of 3,4-diaminobenzoic acid (1a) (4.6 g, 30.0 mmol) in methanol (CH₃OH, 100 mL), thionyl chloride (SOCl_2_, 7.2 mL, 100.0 mmol) was added dropwise slowly. The reaction mixture was stirred at room temperature for 2 hours (reaction completion monitored by TLC). After completion, the mixture was evaporated under reduced pressure to remove the solvent, yielding a crude product. The crude product was recrystallized from cold methanol (20 mL), and the solid was collected by filtration and washed twice with diethyl ether (20 mL) to afford methyl 3,4-diaminobenzoate (**4a**) as a pale yellow solid (4.5 g, 90% yield).

**4b**: To a solution of methyl 3,4-diaminobenzoate (4a) (3.3 g, 20 mmol) in a round-bottom flask, water (H₂O, 20 mL), hydrochloric acid (HCl, 37%, 2 mL), and selenium dioxide (SeO₂, 2.2 g, 20 mmol) were added sequentially. The reaction mixture was stirred at 80ºC for 3 hours. After completion, it was cooled to room temperature and poured into ice water (50 mL). The precipitate was collected by filtration, washed sequentially with cold water (20 mL) and diethyl ether (30 mL), and dried under vacuum to afford methyl benzo[1,2,5]selenadiazole-4-carboxylate (**4b**) as a yellow solid (4.0 g, 85% yield).

**4c:** To a solution of methyl 4b (3.6 g, 15.0 mmol) in ethanol (EtOH, 100 mL), hydrazine hydrate (N₂H₄·H₂O, 80%, 5 mL, 80.0 mmol) was added. The reaction mixture was stirred under reflux overnight. After completion, it was cooled to room temperature and filtered. The solid was washed with ethanol (30 mL) to afford benzo [1,2,5] selenadiazole-4-carbohydrazide (**4c**) as a white solid (2.0 g, 56% yield).

**TSe**: To a solution of **4c** (0.75 g, 3.1 mmol) and **TFB** (0.162 g, 1 mmol) in water (50 mL), the reaction mixture was stirred at room temperature for 1 hour. After completion, the precipitate was collected by filtration and washed sequentially with water (50 mL) and diethyl ether (15 mL). The solid was dried under vacuum for 24 hours to afford the target compound TSe as an orange solid (0.87 g, 95% yield).

**TSe**: Mp = 340.1~342.4 ºC. ^1^H NMR (600 MHz, DMSO-*d*_6_) δ 12.45 – 12.25 (s, 3H), 8.70 – 8.61 (s, 3H), 8.57 – 8.48 (s, 3H), 8.24 – 8.19 (d, *J* = 4.2 Hz, 3H), 8.07 – 7.89 (s, 6H). HRMS (ESI): calcd for [C_30_H_19_N_12_O_3_Se_3_ ]^+^: 834.9199, found: 834.9229.

2. Structural and crystal analysis

**Figure S1.** Molecular structure of **TASe**, conformational isomerization of the acylhydrazone linkage, and crystal structures of the enantiomers. (a) Chemical structure of **TASe** together with the corresponding ^1^H NMR spectrum and peak assignments. The splitting of resonances associated with the acylhydrazone fragment indicates the coexistence in solution of *cis* and *trans* conformational isomers arising from restricted rotation about the amide bonds within the acylhydrazone motifs; the characteristic proton H^g^ on the chiral carbon of the alanine moiety is also assigned. (b) Comparative single-crystal structures of ***L*‑TASe** and ***D*‑TASe**. Both adopt a combined conformational arrangement featuring one *cis* and two *trans* amide conformations, highlighting the conformational correspondence between the two enantiomers in the solid state.

**Figure S2.** Optical micrographs of single crystals of ***L*-TASe** (a) and ***D*-TASe** (b).

**Table S1.** Crystal data and structure refinement for ***L*-TASe** and ***D*-TASe**.

| Compound reference | ***L*-TASe** | ***D*-TASe** |
| --- | --- | --- |
| Empirical formula | C_39_H_33_N_15_O_6_Se_3_ | C_39_H_33_N_15_O_6_Se_3_ |
| Formula weight | 1044.68 | 1044.68 |
| Temperature/K | 100.15 | 100.00(10) |
| Crystal system | orthorhombic | orthorhombic |
| Space group | P2_1_2_1_2 | P2_1_2_1_2 |
| a/Å | 46.6054(7) | 21.4540(5) |
| b/Å | 21.4679(3) | 46.9004(10) |
| c/Å | 4.69900(10) | 4.68060(10) |
| α/º | 90 | 90 |
| β/º | 90 | 90 |
| γ/º | 90 | 90 |
| Volume/Å^3^ | 4701.44(14) | 4709.62(18) |
| Z | 4 | 4 |
| ρ_calc_g/cm^3^ | 1.476 | 1.473 |
| μ/mm^‑1^ | 3.356 | 3.350 |
| F(000) | 2088.0 | 2088.0 |
| Crystal size/mm^3^ | 0.28 × 0.25 × 0.24 | 0.28 × 0.26 × 0.21 |
| Radiation | CuKα (λ = 1.54184) | CuKα (λ = 1.54184) |
| 2θ range for data collection/º | 3.792 to 154.052 | 3.768 to 153.032 |
| Index ranges | -56 ≤ h ≤ 58, -20 ≤ k ≤ 26, -5 ≤ l ≤ 5 | -26 ≤ h ≤ 10, -58 ≤ k ≤ 57, -5 ≤ l ≤ 5 |
| Reflections collected | 44894 | 17128 |
| Independent reflections | 9385 [R_int_ = 0.0521, R_sigma_ = 0.0355] | 8414 [R_int_ = 0.0382, R_sigma_ = 0.0509] |
| Data/restraints/parameters | 9385/30/580 | 8414/0/580 |
| Goodness-of-fit on F^2^ | 1.055 | 1.049 |
| Final R indexes [I>=2σ (I)] | R_1_ = 0.0456, wR_2_ = 0.1185 | R_1_ = 0.0360, wR_2_ = 0.0857 |
| Final R indexes [all data] | R_1_ = 0.0499, wR_2_ = 0.1205 | R_1_ = 0.0490, wR_2_ = 0.0955 |
| Largest diff. peak/hole / e Å^-3^ | 0.69/-0.55 | 0.40/-0.44 |
| Flack parameter | -0.029(8) | -0.024(10) |
| CCDC | 2571398 | 2571397 |

**Figure S3. Crystal structure and intermolecular interactions of *D*-TASe. (a) Crystal-packing view within the *ab* plane, showing intermolecular chalcogen bonds (Se···N, Se···O) and hydrogen bonds (N–H···O, N–H···N). (b) Supramolecular packing along the *c*-axis, primarily driven by a triple N–H···O hydrogen-bonding network.**

**Figure S4.** Hierarchical self-assembly of ***D***-**TASe** into helical supramolecular architectures. Schematic representation of three distinct helical motifs (*P*-, *M*-, and *M*-type) generated through cooperative chalcogen bonds (Se···N, Se···O) and hydrogen bonds (N–H···O, N–H···N). These motifs collectively assemble into a long-range supramolecular helical framework dominated by *M*-type helicity.

**Table S2. Structural parameters and interaction energies of intermolecular interactions in the *L*-TASe crystals and *D*-TASe crystals.**

| Crystal | Intermolecular interactions |
| --- | --- |
| ***L*-TASe** | Se–N^a^…^c’^H–N (*d*_N∙∙∙H_ = 2.45 Å; ∠NHN = 169.6º)  C=O^d’^…^a^H–N (*d*_O∙∙∙H_ = 2.19 Å; ∠NHO = 163.7º)  ^f’’^N–Se…^a’^N–Se (*d*_Se∙∙∙N_ = 2.95 Å; ∠SeNSe = 171.7º)  Se–^a’^N…Se–^f’’^N (*d*_N∙∙∙Se_ = 3.30 Å; ∠NSeN = 160.1º)  ^a’’^N–Se…^d^O=C (*d*_Se∙∙∙O_ = 3.09 Å; ∠NSeO = 168.6º)  C=O^e’’^… ^b’’^H–N (*d*_O∙∙∙H_ = 1.99 Å; ∠NHO = 149.9º)  C=O^e’^…^b’^H–N (*d*_O∙∙∙H_ = 2.13 Å; ∠NHO = 130.3º)  C=O^e^…^b^H–N (*d*_O∙∙∙H_ = 2.17 Å; ∠NHO = 167.6º) |
| ***D*-TASe** | Se–N^a^…^c’^H–N (*d*_N∙∙∙H_ = 2.42 Å; ∠NHN = 170.6º)  C=O^d’^…^a^H-N (*d*_O∙∙∙H_ = 2.19 Å; ∠NHO = 163.5º)  ^f’’^N–Se…^a’^N–Se (*d*_Se∙∙∙N_ = 2.90 Å; ∠NSeN = 171.6º)  Se–^a’^N…Se–^f’’^N (*d*_N∙∙∙Se_ = 3.22 Å; ∠NSeN = 162.5º)  ^a’’^N–Se…^d^O=C (*d*_Se∙∙∙O_ = 3.18 Å; ∠NSeO = 164.1º)  C=O^e’’^…^b’’^H–N (*d*_O∙∙∙H_ = 1.75 Å; ∠NHO = 166.0º)  C=O^e’^…^b’^H–N (*d*_O∙∙∙H_ = 2.12 Å; ∠NHO = 129.7º)  C=O^e^…^b^H–N (*d*_O∙∙∙H_ = 2.20 Å; ∠NHO = 169.4º) |

3. Solid-state characterization of chiral TASe assembly

**Figure S5.** Magnified PXRD patterns of ***L*-TASe** and ***D*-TASe** crystals. The experimental peaks match well with the simulated pattern, confirming phase purity and structural consistency.

**Figure S6.** PXRD patterns of ***L*-TASe** (a) and ***D*-TASe** (b) before and after thermal vacuum treatment. The principal diffraction peaks are largely retained at their original positions, indicating no framework collapse or loss of long-range order.

**Figure S7. FTIR spectra of *L*-TASe crystals after 30-day incubation in aqueous media at pH 2.0 - 7.8. The spectra remained essentially unchanged compared with the as-synthesized sample.**

**4. Solution-phase assembly**

**Figure S8.** Tyndall effect of ***L***-**TASe** (a) and ***D*-TASe** (b) in aqueous solution. Photographs of ***L*-TASe** and ***D*-TASe** aqueous solutions at concentrations ranging from 1 to 10 μM under irradiation with a red laser (DMSO content: 0.05% v/v). The pronounced light scattering along the laser beam path confirms the formation of colloidal aggregates; the scattering intensity increases with concentration, indicating concentration-dependent aggregation.

**Figure S9**. Concentration-dependent emission behavior of ***L*-TASe** in aqueous solution. (a) Emission spectra of ***L*-TASe** at concentrations ranging from 1 to 150 μM, showing an emission maximum at ~705 nm. The emission intensity increased with concentration up to 20 μM and decreased at higher concentrations, consistent with aggregation-induced scattering attenuation. (b) Plot of the emission intensity at 705 nm as a function of ***L*-TASe** concentration. Inset: Photograph of an aqueous ***L*-TASe** dispersion under 365 nm UV light, showing no visible red emission. The apparent signal at 705 nm is therefore attributed to excitation-light scattering by the ***L*-TASe** nanoassemblies, along with the second-order optical response of the spectrometer, rather than to intrinsic molecular fluorescence.

**Figure S10.** DLS size distributions of ***L***-**TASe** assemblies at 5 - 40 μM, showing hydrodynamic diameters ranging from 240 to 280 nm with narrow size distributions.

**Figure S11.** SEM images of ***L*-TASe** nanoassemblies at concentrations ranging from 5 to 100 μM. Well-defined spherical nanoassemblies are observed across all tested concentrations, with surface coverage increasing progressively with concentration.

**Figure S12**. SEM image of ***L*-TASe** nanoassemblies at 20 μM (a) and corresponding particle size distribution determined from SEM image analysis (b). The histogram shows the fraction of particles within different size ranges, with the red line representing the Gaussian fit. The assemblies exhibit a narrow size distribution with an average diameter of ~210 nm, consistent with the DLS results.

**Figure S13.** LD spectra of ***L*-TASe** in aqueous solution and the solid state. Negligible LD signals are observed in both media, with the spectra remaining close to the zero baseline, indicating the absence of appreciable macroscopic anisotropic absorption under the measurement conditions.

**Figure S14.** Chemical stability of ***L*‑TASe** assemblies after 30-day incubation at pH 2.0 - 7.8. The recovered solids were analyzed by ^1^H NMR in DMSO-*d*_6_. The spectra remained essentially unchanged compared with the pristine monomer, indicating structural integrity across a broad pH range.

**Figure S15.** Absorption spectra of supernatants from ***L*-TASe** assemblies after 30-day incubation at pH 2.0 - 7.8. The black curve corresponds to the water-soluble precursor **1d** (Scheme S1) as a reference. No characteristic absorption peaks of the precursor were observed in any supernatant, indicating the absence of detectable decomposition or leaching, and confirming the excellent chemical stability of the assemblies.

**Figure S16.** X‑ray characterization of ***L***-**TASe** assemblies formed in aqueous solution. (a) PXRD pattern of freeze-dried ***L***-**TASe** assemblies, showing only a broad diffuse feature and no sharp Bragg reflections, indicating the absence of long-range crystalline order. (b) Corresponding one-dimensional SAXS profile of ***L***-**TASe** aqueous solution (1 mM) obtained by azimuthal integration after background subtraction, displaying a broad scattering feature indicative of short-range intermolecular correlations. (c) Two-dimensional SAXS pattern of ***L***-**TASe** aqueous solution (1 mM) showing an isotropic diffuse scattering ring after background subtraction, consistent with randomly oriented nanoassembled domains lacking long-range single-crystalline order.

**Figure S17.** SEM images of the six **TASe** control compounds: (a) **PASe**, (b) **TAPh**, (c) **TMASe**, (d) **TPh**, (e) **TSe**, and (f) **TMAPh**. The control compounds exhibit distinct morphologies, including fibrous, sheet-like, film-like, spherical, and irregularly aggregated structures, indicating that the benzoselenadiazole unit, acylhydrazone/alanine motifs, and C_3_-symmetric architecture collectively govern the supramolecular morphology.

**Figure S18.** DLS size distributions of the six control compounds in aqueous solution: (a) **PASe**, (b) **TAPh**, (c) **TMASe**, (d) **TPh**, (e) **TSe**, and (f) **TMAPh**. The main particle-size peaks are approximately 310, 80, 100, 350, 15, and 92 nm, respectively, demonstrating that the structural motifs and molecular topology substantially influence their self-assembly behavior and particle sizes.

**Figure S19.** CD and absorption spectra of L-configured control compounds in aqueous solution: (a) **PASe**, (b) **TAPh**, (c) **TMASe**, (d) **TPh**, (e) **TSe**, and (f) **TMAPh**. Among them, **PASe**, which forms relatively ordered assemblies, exhibits a strong CD signal, whereas **TPh** and **TSe**, lacking the amino acid moiety, show no detectable CD response. The other amino acid-containing compounds display only weak CD signals, further highlighting the essential role of the alanine stereocenters in chirality transfer to the supramolecular level.

5. Antioxidative, cardioprotective and cytotoxic activities

**Figure S20**. DLS size distributions of ***L***-**TASe** assemblies (100 μM) after 2-day incubation in water, DMEM, and DMEM with 1% or 10% FBS. The assemblies retained comparable nanoscale size distributions under all tested conditions, with no pronounced aggregation or size increase, demonstrating their favorable stability under cell culture conditions.

**Figure S21**. Concentration-dependent protective effects of ***L*-TASe** and ***D*-TASe** against DOX-induced H9c2 cytotoxicity. (a, b) Cell viability of H9c2 cells treated with 1 μM DOX and increasing concentrations of ***L*-TASe** (a) or ***D*-TASe** (b). (c, d) Cell viability of H9c2 cells treated with 5 μM DOX and increasing concentrations of ***L*-TASe** (c) or ***D*-TASe** (d). Both enantiomeric assemblies restored cell viability in a concentration-dependent manner under low-dose DOX conditions. Data are presented as mean ± SD from six independent experiments. Statistical significance was determined by one-way ANOVA followed by Tukey’s multiple-comparisons test. n.s., not significant; *P < 0.05, **P < 0.01, ***P < 0.001 versus DOX.

**Figure S22.** Cardioprotective effects of control compounds against DOX-induced H9c2 injury after 48 h co-incubation. Cell viability of H9c2 cells treated with 1 μM DOX and increasing concentrations of structurally related control compounds: **TMAPh** **(a)**, **TMASe** **(b)**, **TPh** **(c)**, **PASe** **(d)**, **TAPh** **(e)**, and **TSe** **(f)**. In contrast to **TASe**, none of the control compounds restored cell viability or exhibited dose-dependent protection, highlighting the structure-specific cardioprotective activity of the **TASe** scaffold. Data are presented as mean ± SD from six independent experiments. Statistical significance was determined by one-way ANOVA followed by Tukey’s multiple-comparisons test. n.s., not significant; *P < 0.05, **P < 0.01, ***P < 0.001 versus DOX.

**Figure S23.** Cardioprotective effects of **TASe** control compounds against DOX-induced H9c2 injury after 48 h co-incubation. Cell viability of H9c2 cells treated with 15 μM DOX and increasing concentrations of structurally related control compounds: **TMAPh** **(a)**, **TMASe** **(b)**, **TPh** **(c)**, **PASe** **(d)**, **TAPh** **(e)**, and **TSe** **(f)**. In contrast to **TASe**, none of the control compounds restored cell viability or exhibited dose-dependent protection under 15 μM DOX challenge, highlighting the structure-specific cardioprotective activity of the **TASe** scaffold. Data are presented as mean ± SD from six independent experiments. Statistical significance was determined by one-way ANOVA followed by Tukey’s multiple-comparisons test. n.s., not significant; *P < 0.05, **P < 0.01, ***P < 0.001 versus DOX.

**Figure S24.** DPPH radical-scavenging activities of ***L***-**TASe**, ***D***-**TASe**, and vitamin C. (a) Representative 96-well plate image showing DPPH decolorization after treatment with ***L***-**TASe**, ***D***-**TASe**, and vitamin C at 1 - 40 μM. Vitamin C exhibited marked concentration-dependent decolorization, whereas ***L***-**TASe** and ***D***-**TASe** showed only weak decolorization. (b-d) Quantitative DPPH radical-scavenging activities of ***L***-**TASe** (b), ***D***-**TASe** (c), and vitamin C (d). Vitamin C served as the positive control. Both ***L***-**TASe** and ***D***-**TASe** showed weak radical-scavenging activity compared with vitamin C. Data are presented as mean ± SD from six independent experiments. Statistical significance was determined by one-way ANOVA followed by Tukey’s multiple-comparisons test. n.s., not significant; *P < 0.05, **P < 0.01, ***P < 0.001 versus 1 μM.

**Figure S25.** Absorption spectra of free DOX (10 μM), ***L*-TASe** assemblies (10 μM), and an ***L*-TASe** + DOX mixture (10 μM each) in aqueous solution. The mixture was incubated at room temperature for 4 h under gentle agitation prior to measurement. Free DOX exhibited a characteristic absorption peak at ~484 nm, whereas ***L*-TASe** showed negligible absorption in this region. The mixture retained the DOX absorption peak at ~484 nm with no significant spectral changes relative to free DOX, indicating no appreciable encapsulation of DOX by ***L*-TASe** assemblies under the tested conditions.

**Figure S26.** Fluorescence microscopy images of intracellular ROS levels in MDA-MB-231 cells under different treatments. Intracellular ROS was stained with DCFH-DA (green), and nuclei were counterstained with Hoechst 33342 (blue). Groups: control, DOX (1 μM), ***L***-**TASe** (8 μM) + DOX (1 μM), and positive control (100 μM H_2_O_2_). Merged images of green and blue channels are shown in the bottom row. Fluorescence intensity reflects intracellular ROS accumulation levels.

**Figure S27. Representative Western blot analysis and corresponding original blot images. (a) Representative Western blot bands showing the expression levels of p-ERK, total ERK, p-Drp1 (Ser616), total Drp1, γ-H2AX, and β-actin in three experimental groups. Western blotting was performed using 15-well SDS-PAGE gels.** **ERK, p-ERK, Drp1, and p-Drp1 (Ser616) were resolved on one gel, whereas γ-H2AX and β-actin were resolved on a second gel. Molecular-weight markers (Thermo Fisher Scientific, Cat. No. 26616-250UL) were loaded at both edges and in the middle lane of the corresponding gel. All samples were obtained from the same batch and processed simultaneously. β-Actin was detected using matched samples from the same experimental groups and on the corresponding membrane. (b) Original Western blot images corresponding to the bands shown in panel (a). The blue and red outlines indicate the original blot images used for the detection of ERK/p-ERK/Drp1/p-Drp1 (Ser616) and γ-H2AX/β-actin, respectively. Molecular-weight markers are shown at the sides of the corresponding blots. Immunoreactive bands were visualized on X-ray film in a darkroom. Band intensities were quantified using ImageJ software. Local background subtraction was performed by subtracting the background intensity measured from the area immediately surrounding each band from the corresponding band intensity. The p-ERK and p-Drp1 (Ser616) signals were normalized to their corresponding total ERK and total Drp1 signals, respectively. Total ERK, total Drp1, and γ-H2AX signals were normalized to β-actin. All Western blot experiments included at least three independent biological replicates. The primary antibodies used were anti-ERK (Proteintech, Cat. No. 66102-1-1g), anti-p-ERK (Cell Signaling Technology, Cat. No. 4370S), anti-Drp1 (Cell Signaling Technology, Cat. No. 8570S), anti-p-Drp1 (Ser616) (Abcam, Cat. No. ab314755), anti-γ-H2AX (Cell Signaling Technology, Cat. No. 9718S), and anti-β-actin (Proteintech, Cat. No. 66009-1-1g).**

**Figure S28.** Densitometric quantification of Western blot analysis. (a) Relative ratio of p-Drp1 (Ser616) to total Drp1. (b) Relative protein level of γ-H2AX normalized to β-actin. (c) Relative protein level of total ERK normalized to β-actin. (d) Relative protein level of total Drp1 normalized to β-actin. **Data are presented as mean ± SD from three independent experiments.** **Statistical significance was determined by one-way ANOVA followed by Tukey’s multiple-comparisons test.** **n.s., not significant; *P < 0.05, **P < 0.01, ***P < 0.001 versus DOX, ^##^P ≤ 0.01, ^###^P ≤ 0.001 versus the control.**

**Table S3.** Comparison of **TASe** with representative cardioprotective systems.

| **Comparative System** | **Typical limitations** | **Distinguishing features of TASe** | **Ref.** |
| --- | --- | --- | --- |
| Conventional DOX nanocarriers | • Therapeutic performance depends entirely on carrier delivery & biodistribution modulation  • Carriers possess no intrinsic cardioprotective capacity  • Uncontrollable drug release, loading efficiency and in vivo distribution weaken therapeutic consistency | • Intrinsic redox-active supramolecular nanosystem  • Alleviates DOX-induced cardiac injury by eliminating ROS, reducing DNA damage and inhibiting ERK overactivation | [2-4] |
| Combination co-administration systems | • Complex formulation preparation; requires precise control of dosing ratio and synchronized drug release  • Antioxidant components may disturb ROS-mediated tumor cytotoxicity in certain cases | • Realizes cardiac protection via simple co-incubation with DOX  • Retains full DOX cytotoxicity toward MDA-MB-231 tumor cells  • Avoids complicated dual-drug co-loading and synchronized release design | [5] |
| Natural product/extract systems | • Complex multicomponent composition with large batch-to-batch variation  • Poor water solubility and weak chemical stability  • Unclear structure–activity relationships  • Cardiochemotherapy interference needs case-by-case verification | • Chemically well-defined C3-symmetric molecular structure  • Clear traceable structure–assembly–activity correlation  • Integrates supramolecular self-assembly and endogenous redox regulatory functions | [6-9] |
| **TASe** assembled system | • Evidence is currently limited to in vitro H9c2 and MDA-MB-231 cell models.  • In vivo efficacy, pharmacokinetics, biodistribution, and long-term safety remain to be established.  • The intracellular fate of the assemblies requires further investigation. | • Single-component integration of self-assembly and ROS-scavenging activity.  • Attenuation of DOX-induced oxidative stress and cellular injury in H9c2 cells.  • No significant reduction in DOX cytotoxicity toward MDA-MB-231 cells under the tested conditions. | This work |

**Figure S29.** Molecular electrostatic potential (MEP) map of ***L*-TASe** calculated at the B3LYP/6-31G(d) level. Red regions denote electron-rich sites (negative potential), while blue regions denote electron-deficient sites (positive potential). The electron-rich areas are predominantly localized around the acylhydrazone heteroatoms, indicating potential sites for ROS interaction.

6. ^1^H NMR, ^13^C NMR and HRMS spectra

**Figure S30.** ^1^H NMR (600 MHz) spectrum of ***L*-TASe** in DMSO-*d*_6_.

**Figure S31.** ^13^C NMR (151 MHz) spectrum of ***L*-TASe** in DMSO-*d*_6_.

**Figure S32.** HRMS (ESI) spectrum of ***L*-TASe**.

**Figure S33.** ^1^H NMR (600 MHz) spectrum of ***D*-TASe** in DMSO-*d*_6_.

**Figure S34.** ^13^C NMR (151 MHz) spectrum of ***D*-TASe** in DMSO-*d*_6_.

**Figure S35.** HRMS (ESI) spectrum of ***D*-TASe**.

**Figure S36.** ^1^H NMR (600 MHz) spectrum of **PASe** in DMSO-*d*_6_.

**Figure S37.** ^13^C NMR (151 MHz) spectrum of **PASe** in DMSO-*d*_6_.

**Figure S38.** HRMS (ESI) spectrum of **PASe**.

**Figure S39.** ^1^H NMR (600 MHz) spectrum of **TAPh** in DMSO-*d*_6_.

**Figure S40.** ^13^C NMR (151 MHz) spectrum of **TAPh** in DMSO-*d_6_*.

**Figure S41.** HRMS (ESI) spectrum of **TAPh**.

**Figure S42.** ^1^H NMR (600 MHz) spectrum of **TMASe** in DMSO-*d*_6_.

**Figure S43.** ^13^C NMR (151 MHz) spectrum of **TMASe** in DMSO-*d*_6_.

**Figure S44.** HRMS (ESI) spectrum of **TMASe**.

**Figure S45.** ^1^H NMR (600 MHz) spectrum of **TMAPh** in DMSO-*d*_6_.

**Figure S46.** ^13^C NMR (151 MHz) spectrum of **TMAPh** in DMSO-*d_6_*.

**Figure S47.** HRMS (ESI) spectrum of **TMAPh.**

**Figure S48.** ^1^H NMR (600 MHz) spectrum of **TPh** in DMSO-*d*_6_.

**Figure S49.** ^13^C NMR (151 MHz) spectrum of **TPh** in DMSO-*d*_6_.

**Figure S50.** HRMS (ESI) spectrum of **TPh**.

**Figure S51.** ^1^H NMR (600 MHz) spectrum of **TSe** in DMSO-*d_6_*.

**Figure S52.** HRMS (ESI) spectrum of **TSe**.

**7. References**

1. K. Lin, P. Weng, Y. Qi, et al., “Evolution of Peptidomimetics-Based Chiral Assemblies of β-Sheet, α-Helix, and Double Helix Involving Chalcogen Bonds,” *ACS Applied Materials & Interfaces 17* (2025): 2863-2870, https://doi.org/10.1021/acsami.4c10568.
2. M. E. R. O’Brien, N. Wigler, M. Inbar, et al., “Reduced cardiotoxicity and comparable efficacy in a phase III trial of pegylated liposomal doxorubicin HCl (CAELYX/Doxil) versus conventional doxorubicin for first-line treatment of metastatic breast cancer.” *Annals of Oncology* *15* (2004): 440-449, https://doi.org/10.1093/annonc/mdh097.
3. M. Najafi, M. R. Hooshangi Shayesteh, K. Mortezaee, et al., “The role of melatonin on doxorubicin-induced cardiotoxicity: A systematic review,” *Life Sciences* *241* (2020): 117173, https://doi.org/10.1016/j.lfs.2019.117173.
4. D. Salas Sanzana, E. Flores Faúndez, J. Meléndez, et al., “Increased delivery and cytotoxicity of doxorubicin in HeLa cells using the synthetic cationic peptide pEM-2 functionalized liposomes,” *Colloids and Surfaces B: Biointerfaces* *228* (2023): 113420, https://doi.org/10.1016/j.colsurfb.2023.113420.
5. H. Dorostkar, B. F. Haghiralsadat, M. Hemati, et al., “Reduction of Doxorubicin-Induced Cardiotoxicity by Co-Administration of Smart Liposomal Doxorubicin and Free Quercetin: In Vitro and In Vivo Studies,” *Pharmaceutics* *15* (2023): 1920, https://doi.org/10.3390/pharmaceutics15071920.
6. Q.-Y. Zhang, F.-X. Wang, K.-K. Jia, et al., “Natural Product Interventions for Chemotherapy and Radiotherapy-Induced Side Effects,” *Frontiers in Pharmacology 9 - 2018* (2018): https://doi.org/10.3389/fphar.2018.01253.
7. A. I. Abushouk, A. Ismail, A. M. A. Salem, et al., “Cardioprotective mechanisms of phytochemicals against doxorubicin-induced cardiotoxicity,” *Biomedicine & Pharmacotherapy* *90* (2017): 935-946, https://doi.org/10.1016/j.biopha.2017.04.033.
8. H. Moutabian, R. Ghahramani-Asl, T. Mortezazadeh, et al., “The cardioprotective effects of nano-curcumin against doxorubicin-induced cardiotoxicity: A systematic review,” *BioFactors* *48* (2022): 597-610, https://doi.org/10.1002/biof.1823.
9. L. Guo, X. Zheng, E. Wang, et al., “Irigenin treatment alleviates doxorubicin (DOX)-induced cardiotoxicity by suppressing apoptosis, inflammation and oxidative stress via the increase of miR-425,” *Biomedicine & Pharmacotherapy* *125* (2020): 109784, https://doi.org/10.1016/j.biopha.2019.109784.
